# Ent2 Governs Morphogenesis and Virulence in Part through Regulation of the Cdc42 Signaling Cascade in the Fungal Pathogen Candida albicans

**DOI:** 10.1128/mbio.03434-22

**Published:** 2023-02-21

**Authors:** Emma Lash, Victoria Prudent, Peter J. Stogios, Alexei Savchenko, Suzanne M. Noble, Nicole Robbins, Leah E. Cowen

**Affiliations:** a Department of Molecular Genetics, University of Toronto, Toronto, Ontario, Canada; b Department of Microbiology and Immunology, UCSF School of Medicine, San Francisco, California, USA; c Department of Chemical Engineering and Applied Chemistry, University of Toronto, Toronto, Ontario, Canada; d Department of Microbiology, Immunology and Infectious Diseases, Cumming School of Medicine, University of Calgary, Calgary, Alberta, Canada; e Center for Structural Genomics of Infectious Diseases (CSGID), Chicago, Illinois, USA; Geisel School of Medicine at Dartmouth

**Keywords:** *Candida albicans*, Cdc42, ENTH, Ent2, epsin, fungal pathogen, morphogenesis, virulence

## Abstract

The ability to transition between yeast and filamentous growth states is critical for virulence of the leading human fungal pathogen Candida albicans. Large-scale genetic screens have identified hundreds of genes required for this morphological switch, but the mechanisms by which many of these genes orchestrate this developmental transition remain largely elusive. In this study, we characterized the role of Ent2 in governing morphogenesis in C. albicans. We showed that Ent2 is required for filamentous growth under a wide range of inducing conditions and is also required for virulence in a mouse model of systemic candidiasis. We found that the epsin N-terminal homology (ENTH) domain of Ent2 enables morphogenesis and virulence and does so via a physical interaction with the Cdc42 GTPase-activating protein (GAP) Rga2 and regulation of its localization. Further analyses revealed that overexpression of the Cdc42 effector protein Cla4 can overcome the requirement for the ENTH-Rga2 physical interaction, indicating that Ent2 functions, at least in part, to enable proper activation of the Cdc42-Cla4 signaling pathway in the presence of a filament-inducing cue. Overall, this work characterizes the mechanism by which Ent2 regulates hyphal morphogenesis in C. albicans, unveils the importance of this factor in enabling virulence in an *in vivo* model of systemic candidiasis and adds to the growing understanding of the genetic control of a key virulence trait.

## INTRODUCTION

The fungal kingdom is renowned for its vast morphological complexity and plasticity, with many species undergoing developmental transitions in response to diverse environmental cues. The ability to switch morphogenetic states occurs for a variety of purposes, including for sexual reproduction, nutrient acquisition, and colonization or establishment of infection (1, 2). Invasive fungal diseases have a devastating impact on human health worldwide, with the fungal pathogen Candida albicans reigning as a leading cause of systemic fungal infections (3). C. albicans is a commensal member of the human mucosal microbiota, but when the opportunity presents itself, such as during host immunosuppression, it can escape from its commensal niche and enter the bloodstream. Once circulating in the blood, C. albicans can invade other internal organs such as the kidney and liver, resulting in deep-seated infections associated with mortality rates up to 40% (3, 4). Its ability to thrive within and spread throughout multiple host tissues is largely due to its developmental plasticity, which allows C. albicans to switch between distinct morphological states (1, 5). In its yeast form, C. albicans can disseminate throughout the bloodstream, while in its elongated filamentous form, C. albicans can secrete virulence factors such as aspartic proteases and a peptide toxin that help it penetrate deep into host tissues (6, 7). Thus, morphogenesis is a key virulence trait in this important human fungal pathogen (1, 8).

A wide range of host-relevant growth conditions, including serum, elevated CO_2_, and nutrient starvation, induce C. albicans to undergo filamentous growth (2, 5). Most inducing conditions require a concurrent temperature increase to 37°C, and temperatures above 39°C are sufficient to induce filamentation independently (2). C. albicans relies on complex and interconnected signaling pathways to regulate its morphology in response to these diverse environmental stimuli. Two of the major signaling pathways that control the filamentous growth program are the cyclic AMP (cAMP) protein kinase A (PKA) pathway and the mitogen-activated protein kinase (MAPK) pathway (2). Most filament-inducing cues signal through the cAMP-PKA pathway, which involves activation of the adenylyl cyclase Cyr1 to generate cAMP (2). The resulting increase in intracellular cAMP levels activates the PKA complex, leading to phosphorylation of transcription factors such as Efg1 and upregulation of filament-specific transcripts (2). Genetic perturbation of the cAMP-PKA pathway by deleting its various components blocks filamentation in response to many cues, including serum, physiological CO_2_, and elevated temperature, as well as impairs virulence in mouse models of infection (2, 9, 10) The MAPK pathway involves activation of the GTPase Cdc42, which signals to its downstream effector kinase, Cst20, to activate a MAPK cascade involving Ste11, Hst7, and Cek1/Cek2, which ultimately activates the transcription factor Cph1 (2). In contrast to the cAMP-PKA pathway, many components of the MAPK pathway are dispensable for filamentation under select inducing cues (2, 11). Notably, Cdc42 can also activate an alternative downstream kinase, Cla4, which is involved in septin organization and is required for filamentation under most inducing cues (5). Overall, complex genetic circuitry involving multiple signaling pathways allows C. albicans to sense and respond to diverse environmental conditions.

Functional genomic screens have been instrumental in dissecting the complex pathways that govern morphogenesis (12). Multiple mutant libraries have been constructed for C. albicans, and screening them under different inducing cues has allowed for the discovery of hundreds of genes encoding morphogenetic regulators (8, 13–16). One of the largest mutant libraries in C. albicans is the Gene Replacement and Conditional Expression (GRACE) collection, which consists of 3,193 tetracycline-repressible mutants covering approximately 50% of the genome (17, 18). Screening of this library has facilitated the discovery of C. albicans essential genes (15, 18), genes involved in regulating drug susceptibility phenotypes (19), and genes required for morphogenesis under diverse filament-inducing cues (15, 16). Large-scale screening results provide an important resource for the field and have facilitated the characterization of novel processes involved in morphogenesis (20). However, many genes identified by these screens have yet to be characterized mechanistically, and thus, our understanding of the genetic underpinnings of this key virulence trait remains incomplete. *ENT2* is one such gene that was identified as required for morphogenesis in response to multiple inducing cues (15, 16, 21); however, the mechanism by which it enables morphogenesis remains enigmatic.

Here, we characterize Ent2 as a central regulator of C. albicans morphogenesis and virulence. We establish that the filamentation function of Ent2 resides within its epsin N-terminal homology (ENTH) domain and requires two specific amino acid residues, Y100 and T104, which we visualize through determination of the crystal structure of this domain. We show that the ENTH domain has an important role in regulating endocytosis and also enables filamentation through a physical interaction with Rga2, the GTPase-activating protein (GAP) for Cdc42. Furthermore, overexpression of the downstream Cdc42 effector, Cla4, overcomes the requirement for the ENTH-Rga2 interaction to initiate hyphal morphogenesis. Finally, we highlight that Ent2, and more specifically, the ENTH domain, is required for virulence in a mouse model of systemic candidiasis. Overall, this work highlights how we can harness knowledge from functional genomic screens to provide mechanistic insights into the core genetic circuitry governing morphogenesis and virulence.

## RESULTS

### Ent2 is a general regulator of filamentous growth in C. albicans.

**FIG 1:**
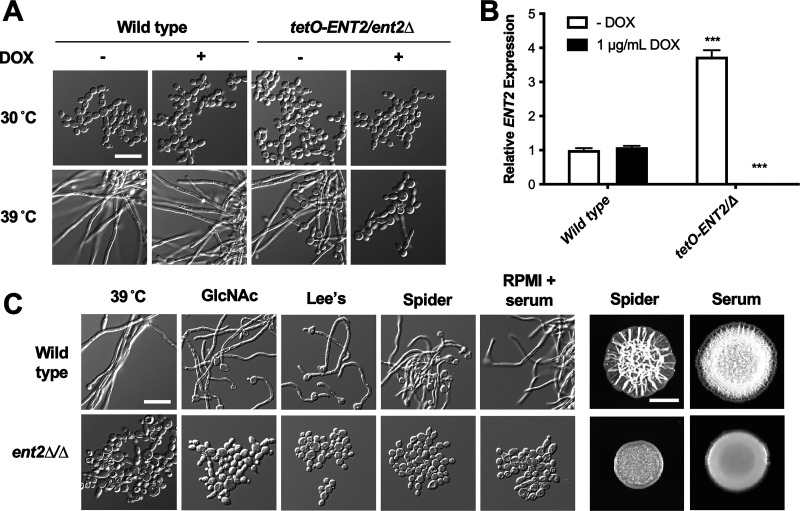
Ent2 is required for filamentation under diverse filament-inducing cues. (A) Genetic depletion of *ENT2* blocks filamentation during growth at 39°C. Cells were grown at the indicated temperature in YPD medium with or without 1 μg/mL doxycycline (DOX) with shaking and imaged at 5 h. Scale bar, 20 μm. (B) Relative *ENT2* expression in wild-type and *tetO-ENT2/ent2Δ* cells in the absence and presence of 1 μg/mL DOX as determined by qRT-PCR, using *ACT1* and *GPD1* for normalization. Values shown are relative *ENT2* expression compared to the wild-type strain in the absence of DOX. Error bars represent SEM above and below the mean of technical triplicates (two-way analysis of variance [ANOVA], Bonferroni correction for multiple comparisons; ***, *P < *0.0001 compared to wild-type untreated). (C) Homozygous deletion of *ENT2* blocks filamentation in response to diverse filament-inducing cues. For liquid conditions, cells were grown in the indicated conditions and imaged at 5 h. All liquid conditions were done with shaking. Scale bar, 20 μm. For solid conditions, cells were spotted onto agar plates and imaged after 72 h of incubation. Scale bar, 5 mm. All cells were incubated at 37°C except where indicated.

Previous work implicated the epsin Ent2 as being important for growth, cell wall homeostasis, efficient endocytosis, and filamentation upon exposure to serum as well as in mammalian tissue culture media (solid and liquid RPMI 1640 and solid M199) (15, 21). We validated this gene was important for filamentation using the *ENT2* strain from the GRACE library (17). In this mutant, one allele of *ENT2* is deleted, and the remaining wild-type allele is under the control of a tetracycline-repressible (*tetO*) promoter that is repressed by the addition of doxycycline (DOX). We probed the capacity of *tetO-ENT2/ent2Δ* cells to filament during growth at 39°C, a robust filament-inducing cue that mimics febrile episodes in the host. In the absence of DOX, the *tetO-ENT2/ent2Δ* strain underwent robust filamentation at 39°C (Fig. 1A). However, upon transcriptional repression of *ENT2* by the addition of 1 μg/mL DOX (Fig. 1B), this strain was unable to form filaments at 39°C and instead continued to proliferate as yeast (Fig. 1A).

The developmental plasticity of C. albicans to grow in either the yeast or filamentous state allows it to successfully colonize multiple sites within the human host. To respond appropriately to its environment, C. albicans has evolved multiple independent and overlapping signaling pathways that sense and respond to a wide range of inducing stimuli (5). As such, genes required for filamentation in response to one inducing cue present in a particular growth medium may be entirely dispensable in response to another (22). Therefore, we wanted to determine whether Ent2 is a general regulator of filamentation or is only required for filamentation under a narrower range of inducing cues. For these experiments, we deleted both copies of *ENT2* in the standard laboratory SN95 background using a transient CRISPR method (23). The *ent2Δ/ent2Δ* mutant phenocopied the *tetO-ENT2*/*ent2Δ* strain with DOX and was unable to form filaments in 1% yeast extract, 2% peptone, and 2% glucose (YPD) medium at 39°C (Fig. 1C). Furthermore, the *ent2Δ/ent2Δ* mutant was blocked in filamentation when grown at 37°C in the presence of 5 mM *N*-acetylglucosamine (GlcNAc), Lee’s medium, Spider medium, and RPMI supplemented with 10% serum under shaking conditions (Fig. 1C). As well, the *ent2Δ/ent2Δ* mutant could not form wrinkly colonies on Spider or serum agar after 72 h of incubation at 37°C (Fig. 1C), a phenotype that often correlates with the extent of filamentous growth of individual cells (24). Thus, Ent2 is a general regulator of filamentous growth in C. albicans.

### The ENTH domain of C. albicans Ent2 mediates filamentation.

**FIG 2:**
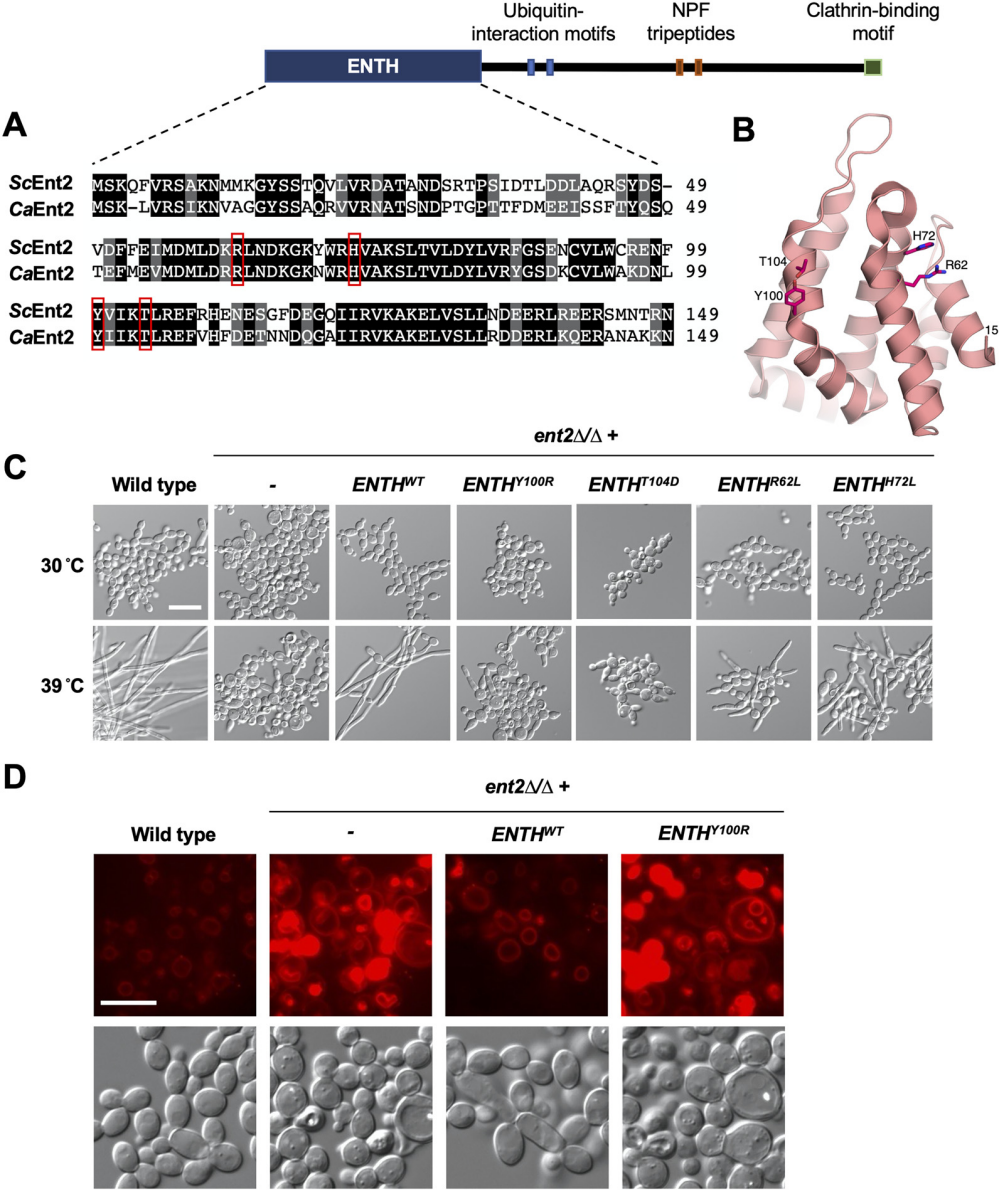
The C. albicans epsin N-terminal homology (ENTH) domain of Ent2 is required for filamentation. (A) Domain organization of C. albicans Ent2 and Smith-Waterman pairwise alignment of ENTH domain from S. cerevisiae Ent2 and C. albicans Ent2. Conserved residues prioritized for site-directed mutagenesis (SDM) experiments are indicated in red boxes. (B) Crystal structure of C. albicans Ent2 ENTH domain. The four residues R62, H72, Y100, and T104 are shown in stick representation. (C) Reintroduction of a single copy of a wild-type ENTH domain or R62L or H72L mutant domains restores filamentation in an *ent2Δ/ent2Δ* background. Reintroduction of a single copy of Y100R or T104D mutant domains in an *ent2Δ/ent2Δ* background does not restore filamentation. Cells were grown at the indicated temperature in YPD medium for 5 h with shaking. Scale bar, 20 μm. (D) Microscopy images of wild type and *ent2Δ/ent2Δ* mutants grown at 30°C and incubated with FM4-64. Scale bar, 10 μm.

Epsin proteins are highly conserved among eukaryotes. Through the full-length protein sequences, C. albicans Ent2 (CaEnt2) shares 40 to 43% identity and 51 to 56% similarity with its Saccharomyces cerevisiae homologs ScEnt2 and ScEnt1. Similar to its S. cerevisiae homologs and other epsins, CaEnt2 has a ~150-amino-acid-long ENTH domain, followed by two ubiquitin-interaction motifs for cargo binding, two Asn-Pro-Phe (NPF) tripeptides for interactions with other endocytic machinery, and a C-terminal clathrin binding motif (21, 25–27) (Fig. 2A). To understand the molecular function of CaEnt2, we solved the crystal structure of the C. albicans ENTH domain (Fig. 2B; see Table S1 in the supplemental material). The structure revealed the canonical ENTH structure composed of six ɑ-helices forming a compact globular structure, with the two C-terminal helices (residues 89 to 108 and 119 to 149) containing kinks bending them toward the core of the fold (Fig. 2B). Superposition of the ENTH domains from the structures of CaEnt2 and ScEnt2 in complex with phosphatidylinositol 4,5-bisphosphate (PIP2; PDB accession no. 5ON7) (28) revealed nearly identical structures, with a root mean square deviation (RMSD) of 0.7 Å over 131 matching Cɑ residues, with 61% sequence identity and 78% similarity (Fig. 2A; Fig. S1). There were no notable structural differences between these two proteins except for the fact that in our structure of CaEnt2, the PIP2 binding site was not fully resolved in the electron density. In particular, the N-terminal residues 1 through 14 of CaEnt2 are not ordered in our structure, and we did not identify a dimeric arrangement as was observed for the PIP2 binding competent form of ScEnt2. These observations can be attributed to the absence of the lipid headgroup in our crystallization experiments. We did, however, observe conservation of the residues R62, H72, Y100, and T104, which are involved in PIP2 binding and/or protein-protein interactions in ScEnt2 (25). These striking observations of sequence and structural conservation led us to further investigate the function of the ENTH domain in regulating Ent2 functions in C. albicans, using the function of the ScEnt2 protein as a guide.

10.1128/mbio.03434-22.1 FIG S1 Comparison of the structures of ENTH domains from CaEnt2 and ScEnt2. CaEnt2 is shown in pink, and the ScENT2-PIP2 complex (PDB accession no. 5ON7) (28) is shown as two shades of blue for the dimeric ligand-bound form. Residues R62, H72, Y100, and T104 are shown as sticks. PIP2 is shown as ball and stick. Residues 1 and 15 are indicated. Download FIG S1, TIF file, 2.7 MB. Copyright © 2023 Lash et al. 2023 Lash et al. https://creativecommons.org/licenses/by/4.0/ This content is distributed under the terms of the Creative Commons Attribution 4.0 International license.

10.1128/mbio.03434-22.7 TABLE S1 X-ray crystallographic statistics. Download Table S1, DOCX file, 0.01 MB. Copyright © 2023 Lash et al. 2023 Lash et al. https://creativecommons.org/licenses/by/4.0/ This content is distributed under the terms of the Creative Commons Attribution 4.0 International license.

S. cerevisiae *ENT1* and *ENT2* are each nonessential, but deletion of both is synthetically lethal (25). A single ENTH domain is sufficient to restore viability in S. cerevisiae *ent1Δ ent2Δ* double mutants, indicating that the ENTH domain harbors the essential function of this protein (25). Therefore, we hypothesized that the ENTH domain of C. albicans Ent2 may be the critical functional unit for filamentation. To test this, we reintroduced a single copy of the wild-type CaEnt2 ENTH domain (ENTH^WT^) at the native locus in the *ent2Δ/ent2Δ* mutant. This *ent2Δ/ent2Δ* plus ENTH^WT^ strain was capable of filamenting at 39°C to a similar degree as the wild-type parental control (Fig. 2C), revealing that Ent2 mediates filamentation through its ENTH domain.

To further explore how the ENTH domain promotes filamentation, we investigated the importance of highly conserved residues in regulating this important virulence trait by performing site-directed mutagenesis. In S. cerevisiae, expression of the ScENTH1 domain harboring either a Y100R or T104D substitution is unable to restore viability in *ent1Δ ent2Δ* mutants (26). Similarly, we found that neither CaENTH^Y100R^ nor CaENTH^T104D^ could restore filamentation in the absence of Ent2 in C. albicans (Fig. 2C) despite adequate protein expression (Fig. S2). Additionally, *ent2Δ/ent2Δ* cells harboring the ENTH^Y100R^ domain did not upregulate the filament-specific transcripts *HWP1* or *ALS3* to the same degree as those harboring the ENTH^WT^ domain when grown at 39°C (Fig. S3). Next, we investigated the residues R62 and H72, which facilitate lipid binding at the plasma membrane (26). In contrast to the Y100 and T104 substitutions, ENTH domains harboring substitutions for either of these lipid-binding residues still partially complement the *ent1Δ ent2Δ* cell viability defect in S. cerevisiae (26). Accordingly, reintroducing either ENTH^R62L^ or ENTH^H72L^ in C. albicans partially restored filamentation in the *ent2Δ/ent2Δ* background (Fig. 2C). Overall, these results indicate that C. albicans Ent2 regulates filamentation through its ENTH domain, and specific residues important for protein binding are required for this function, but lipid-binding residues appear to be dispensable.

10.1128/mbio.03434-22.2 FIG S2 The C. albicans ENTH^Y100R^ and ENTH^T104D^ domains that do not restore filamentation are expressed. Western blotting was performed on cell lysates from *ent2Δ/ent2Δ* cells expressing ENTH^WT^-His_6_-FLAG_3_, ENTH^Y100R^-His_6_-FLAG_3_, or ENTH^T104D^-His_6_-FLAG_3_ with an anti-FLAG antibody to detect ENTH. Wild type serves as untagged control. Coomassie-stained membrane serves as protein loading control. Download FIG S2, TIF file, 0.7 MB. Copyright © 2023 Lash et al. 2023 Lash et al. https://creativecommons.org/licenses/by/4.0/ This content is distributed under the terms of the Creative Commons Attribution 4.0 International license.

10.1128/mbio.03434-22.3 FIG S3 The wild-type ENTH domain is required for hyphal-specific gene expression during growth at 39°C. Relative *HWP1* (A) and *ALS3* expression (B) in *ent2Δ/ent2Δ* plus *ENTH^WT^* and *ent2Δ/ent2Δ* plus *ENTH^Y100R^* cells grown at 30°C and 39°C by qRT-PCR, using *ACT1* and *GPD1* for normalization. Values shown at relative *HWP1* or *ALS3* expression compared to *ent2Δ/ent2Δ* plus *ENTH^WT^* at 30°C. Error bars represent SEM above and below the mean of technical triplicates (two-way ANOVA, Bonferroni correction for multiple comparisons; ****, *P < *0.0001; *, *P < *0.05). Download FIG S3, TIF file, 1.0 MB. Copyright © 2023 Lash et al. 2023 Lash et al. https://creativecommons.org/licenses/by/4.0/ This content is distributed under the terms of the Creative Commons Attribution 4.0 International license.

Although the protein motifs that are known to contribute to endocytosis reside outside the ENTH domain, including the NPF tripeptides and clathrin binding motif, we tested whether the ENTH domain was also important for endocytosis. For this, we used two dyes that mark endocytosis, FM4-64 and Lucifer yellow (21, 29). We found that *ENT2* is required for efficient endocytosis, as homozygous deletion resulted in heterogenous staining, with some cells displaying increased signal intensity and reduced endocytic and vacuole membrane staining (Fig. 2D; Fig. S4). Interestingly, reintroducing a single copy of the ENTH^WT^ domain restored endocytosis to a similar extent as that observed with a wild-type control (Fig. 2D; Fig. S4). However, reintroduction of the ENTH^Y100R^ domain did not restore endocytosis and, instead, mimicked complete loss of function of Ent2 (Fig. 2D; Fig. S4). This indicates that the ENTH domain plays a role in regulating endocytosis, which could contribute to its role in filamentation.

10.1128/mbio.03434-22.4 FIG S4 The ENTH domain is important for normal endocytosis. Microscopy images of wild type and *ent2* mutants grown at 30°C and incubated with Lucifer yellow. Scale bar, 20 μm. Download FIG S4, TIF file, 2.2 MB. Copyright © 2023 Lash et al. 2023 Lash et al. https://creativecommons.org/licenses/by/4.0/ This content is distributed under the terms of the Creative Commons Attribution 4.0 International license.

### ENTH physically interacts with Rga2.

The GTPase Cdc42 is a central regulator of cell polarity and controls many aspects of filamentous growth in C. albicans (30). During filamentation, Cdc42 localizes to hyphal tips (31) where it interacts with several proteins involved in the organization of actin, formation of septin, and regulation of exocytosis (5). Cdc42 also signals to two kinases, Cst20 and Cla4, which activate additional signaling cascades important for filamentation (32, 33). As a GTPase, Cdc42 cycles between an active GTP-bound form and an inactive GDP-bound form, and this process is regulated by several proteins. Its guanine nucleotide exchange factor (GEF), Cdc24, promotes Cdc42 activation by catalyzing its binding to GTP. Its GTPase-activating proteins (GAPs), Rga2 and Bem3, promote Cdc42 inactivation by catalyzing the hydrolysis of GTP to GDP via their GAP domain. As such, these GAPs are negative regulators of filamentation, and various cellular processes downregulate their activity in the presence of a filament-inducing cue (34, 35).

**FIG 3:**
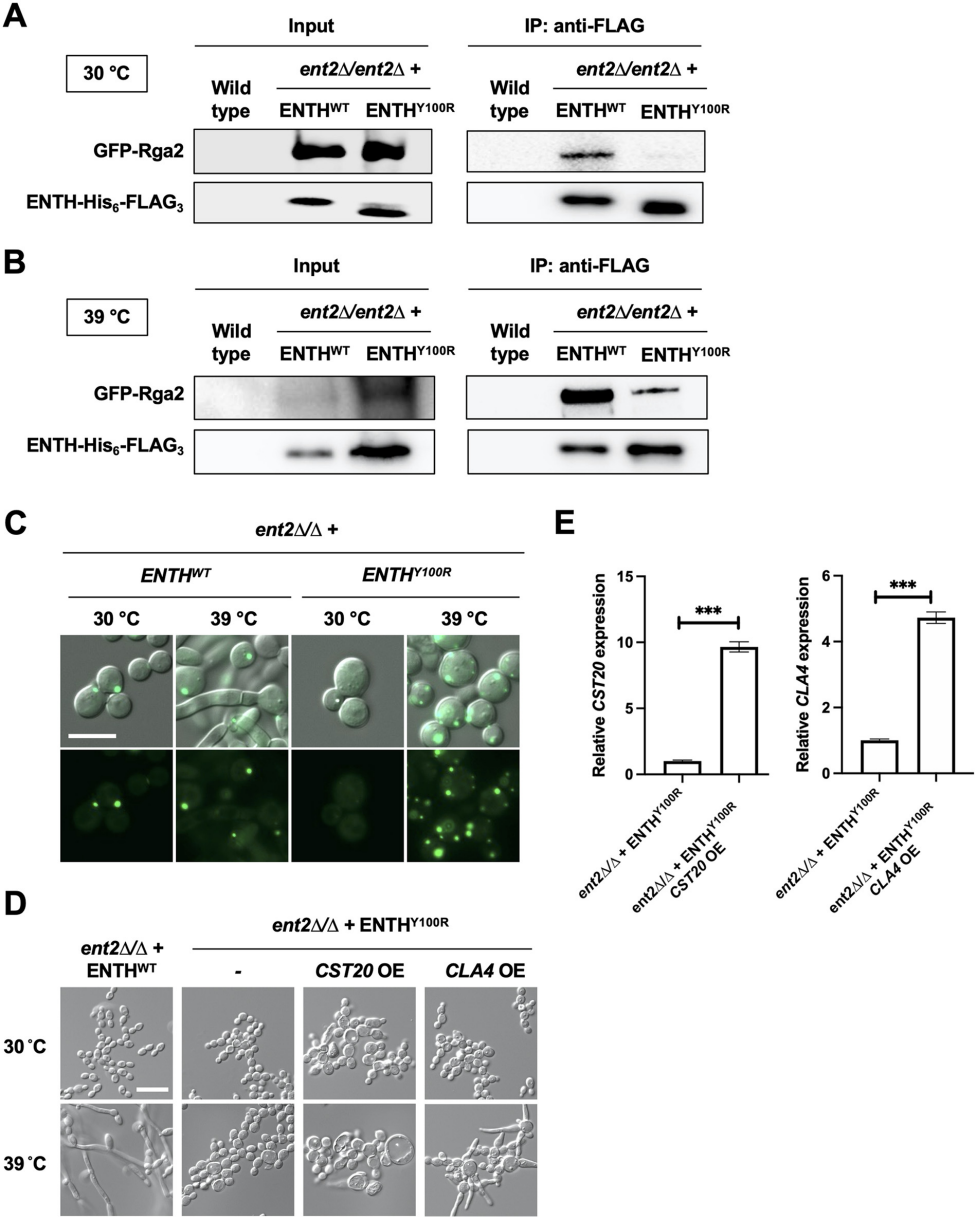
The C. albicans ENTH domain regulates Cdc42 signaling via a physical interaction with Rga2. (A) GFP-Rga2 was pulled down by anti-FLAG beads in *ent2Δ/ent2Δ* cells grown at 30°C expressing ENTH^WT^-His_6_-FLAG_3_ but not ENTH^Y100R^-His_6_-FLAG_3_. Western blotting was performed with an anti-FLAG antibody for ENTH and an anti-GFP antibody for Rga2. The wild type serves as an untagged control. (B) Same as in panel A, but cells were grown at 39°C. (C) GFP-Rga2 was visualized in *ent2Δ/ent2Δ* cells expressing ENTH^WT^ or ENTH^Y100R^ at 30°C or 39°C. Shown are cells expressing GFP-Rga2, as visualized by fluorescence microscopy and DIC images. Scale bar indicates 10 μm. (D) Filamentation defect of the *ent2Δ/ent2Δ* plus *ENTH^Y100R^* strain is partially rescued by overexpression of Cdc42 effector *CLA4* but not by overexpression of *CST20.* Cells were grown at the indicated temperature in YPD medium for 7 h with shaking. OE indicates gene being overexpressed. Scale bar, 20 μm. (E) Overexpression of *CST20* and *CLA4* was determined by qRT-PCR, using *ACT1* and *GPD1* expression for normalization. Values shown are relative *CST20* or *CLA4* expression compared to the parental *ent2Δ/ent2Δ* strain. Error bars represent SEM above and below the mean of technical triplicates (unpaired *t* test; ***, *P < *0.0001).

**FIG 4:**
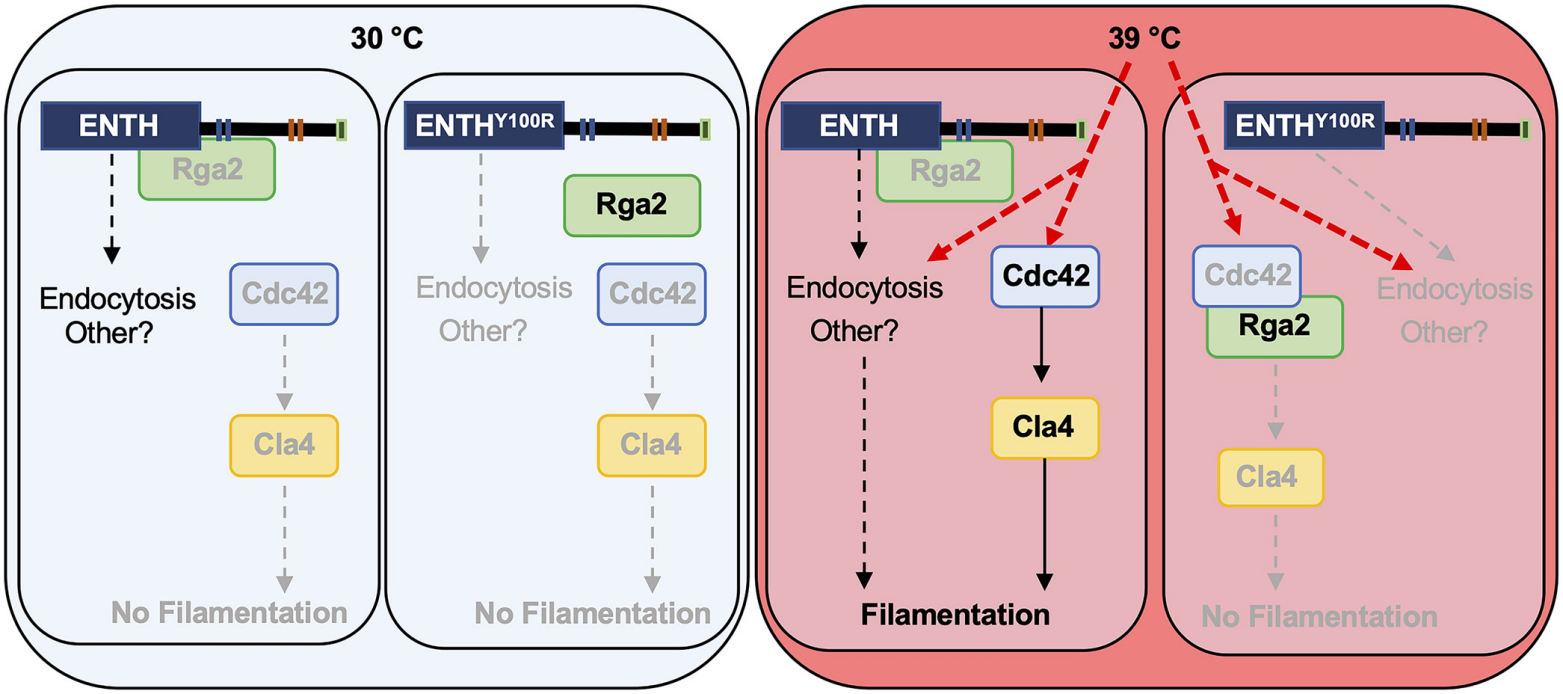
Proposed model for the role of Ent2 in regulating filamentation. Ent2 physically interacts with Rga2 via its ENTH domain, and this interaction requires the Y100 residue. We propose that in the presence of a filament-inducing cue, such as high temperature, Ent2 binds Rga2 such that cellular signaling can activate Cdc42 and downstream effectors such as Cla4 to allow for filamentation. The ENTH domain also regulates endocytosis and other processes, which collectively likely influence filamentation. In the ENTH^Y100R^ domain mutant that cannot physically interact with Rga2, Rga2 continues to inhibit signaling through Cdc42 despite the presence of an inducing cue, resulting in a block in filamentation. Furthermore, endocytosis and other processes downstream of Ent2 are not properly regulated.

S. cerevisiae Ent1 and Ent2 bind Rga2 with two highly conserved residues within the Ent1 ENTH domain, Y100 and T104, required for the ENTH-Rga2 interaction (26). However, CaRga2 and ScRga2 share only 26% identity and 40% similarity. Therefore, we wanted to determine whether this interaction is conserved in C. albicans and, if so, whether the ENTH mutations that abolish filamentation also abolish this interaction. For simplicity in downstream analyses, we proceeded with the *ent2Δ/ent2Δ* plus ENTH^Y100R^ mutant given that the *ent2Δ/ent2Δ* plus ENTH^T104D^ strain possessed an identical filament-defective phenotype. We performed coimmunoprecipitation experiments in *ent2Δ/ent2Δ* cells expressing GFP-Rga2 and either ENTH^WT^-His_6_-FLAG_3_ or ENTH^Y100R^-His_6_-FLAG_3_. Lysates from cells grown at 30°C and 39°C were incubated with anti-FLAG beads, and the pulldown fraction was probed with an anti-FLAG antibody for detection of the ENTH domain and an anti-green fluorescent protein (GFP) antibody for detection of Rga2. At both temperatures, GFP-Rga2 was detected in the strain possessing ENTH^WT^ and greatly reduced in the strain possessing ENTH^Y100R^, indicating a physical interaction between the ENTH domain and Rga2 in yeast and filament conditions that requires Y100 (Fig. 3A and B). Furthermore, these data support the model that the ENTH domain regulates filamentation through a physical interaction with Rga2 (Fig. 4).

Next, we sought to determine the functional relevance of the ENTH-Rga2 association. We used microscopy to assess whether the lack of physical interaction between ENTH^Y100R^ and Rga2 impacted the localization of Rga2. At 30°C in the ENTH^WT^ background, GFP-Rga2 localized to bud tips and bud necks as expected for wild-type yeast (34) (Fig. 3C; Fig. S5). Interestingly, in the ENTH^Y100R^ background at 30°C, the GFP-Rga2 signal was low and diffuse throughout the cytosol, and no punctate localization at bud sites was observed (Fig. 3C; Fig. S5). At 39°C in the ENTH^WT^ background, GFP-Rga2 was localized to bud sites in cells growing as yeast and rarely seen localized to hyphae, as expected (34) (Fig. 3C; Fig. S5). In contrast, GFP-Rga2 formed multiple foci per cell at aberrant locations in the ENTH^Y100R^ mutant background at 39°C (Fig. 3C; Fig. S5). Overall, this indicates that the ENTH-Rga2 physical interaction is important for proper Rga2 localization.

10.1128/mbio.03434-22.5 FIG S5 The ENTH domain is important for proper Rga2 localization. GFP-Rga2 was visualized in *ent2Δ/ent2Δ* cells expressing ENTH^WT^ or ENTH^Y100R^ at 30°C or 39°C. Shown are cells expressing GFP-Rga2, as visualized by fluorescence microscopy and DIC images. Scale bar indicates 20 μm. Download FIG S5, TIF file, 1.9 MB. Copyright © 2023 Lash et al. 2023 Lash et al. https://creativecommons.org/licenses/by/4.0/ This content is distributed under the terms of the Creative Commons Attribution 4.0 International license.

### *CLA4* overexpression partially overcomes the requirement for ENTH-Rga2 interaction.

Given the critical role of the ENTH domain in regulating filamentation as well as endocytosis (Fig. 2D; Fig. S4), we wanted to next assess whether it regulates filamentation through its interaction with Rga2 or if its role in this cellular transition is more complex. We found that genetic depletion of *RGA2* did not restore filamentation in the ENTH^Y100R^ mutant (Fig. S6A and B). However, literature suggesting that epsins regulate RhoGTPase signaling (26, 36), together with our data showing that the ENTH domain binds Rga2 and regulates its localization, prompted us to further probe this pathway. Rga2 negatively regulates Cdc42, preventing signaling to downstream effectors that promote filamentation (35). Therefore, we reasoned that the physical interaction between the ENTH domain and Rga2 inhibits the inactivation of Cdc42, which ultimately enables filamentous growth (Fig. 4). Cdc42 activates the mitogen-activated protein kinase (MAPK) signaling cascade through Cst20, which results in activation of the transcription factor Cph1 and hyphal-specific gene expression. However, this signaling pathway is dispensable under all filament-inducing conditions apart from solid Spider medium (5). The other kinase that functions downstream of Cdc42 is Cla4, which is involved in septin organization and is required for filamentation under most inducing cues (5). To decipher which genetic pathway Ent2 signals through to induce filamentous growth, we generated strains that overexpress either *CST20* or *CLA4* in the filament-defective *ent2Δ/ent2Δ* plus ENTH^Y100R^ background based on the notion that overexpression of downstream regulators should restore filamentation in strains lacking a functional Ent2. Overexpression of *CST20* did not restore filamentation in the *ent2Δ/ent2Δ* plus ENTH^Y100R^ background and instead resulted in sick and enlarged cells (Fig. 3D and E). In contrast, overexpression of *CLA4* partially restored filamentation in the *ent2Δ/ent2Δ* plus ENTH^Y100R^ strain (Fig. 3D and E), suggesting that Cla4 acts downstream of Ent2 to contribute to hyphal morphogenesis (Fig. 4).

10.1128/mbio.03434-22.6 FIG S6 Transcriptional repression of *RGA2* does not bypass the filamentation defect of the ENTH^Y100R^ mutant. (A) Cells were grown in the presence of 50 μg/mL DOX at 30°C or 39°C for 5 h and imaged using DIC microscopy. Scale bar, 20 μm. (B) Indicated cells were grown in the absence or presence of 50 μg/mL DOX at 30°C for 5 h and imaged. DIC microscopy is shown at the top, and GFP is shown at the bottom. Absence of GFP signal in the presence of 50 μg/mL DOX indicates *RGA2* is sufficiently repressed by this concentration of DOX. Scale bar, 20 μm. Download FIG S6, TIF file, 1.7 MB. Copyright © 2023 Lash et al. 2023 Lash et al. https://creativecommons.org/licenses/by/4.0/ This content is distributed under the terms of the Creative Commons Attribution 4.0 International license.

### Ent2 and the ENTH domain are required for virulence in a mouse model of systemic candidiasis.

**FIG 5:**
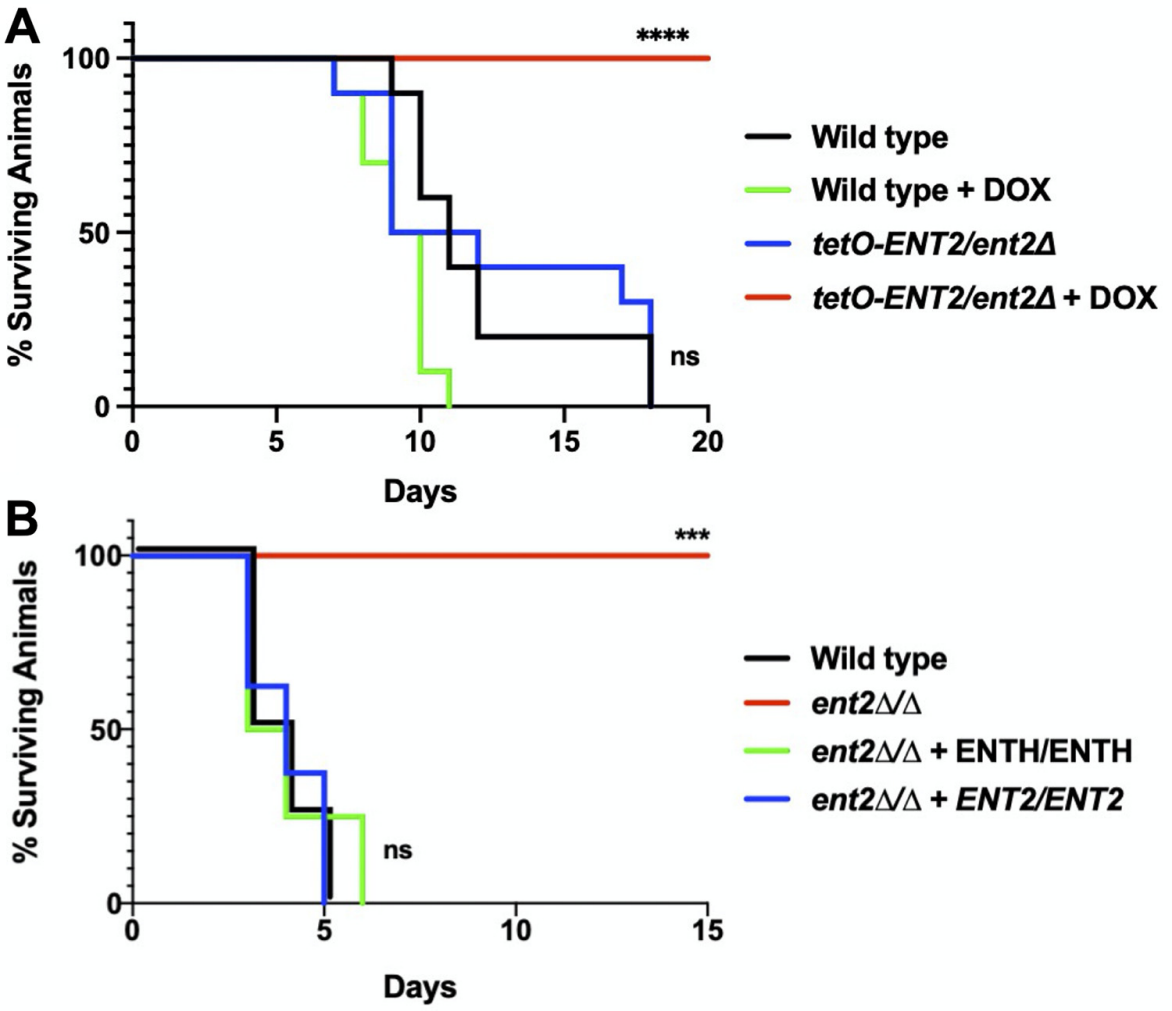
Ent2 regulates virulence through its ENTH domain in a mouse model of systemic candidiasis. (A) Mice were infected with 2 × 10^5^ CFU of wild-type (CaSS1) or *tetO-ENT2/ent2Δ* strains and supplied DOX or solvent in the drinking water to modulate *ENT2* expression. All mice infected with *tetO-ENT2/ent2Δ* and supplied with DOX in the drinking water survived until the end of the experiment. Log-rank (Mantel-Cox) test; ****, *P < *0.0001. Log-rank test for trend, *P = *0.0156. Ten mice were infected per group. (B) Mice were infected with 3 × 10^5^ CFU of either wild type (SN95), *ent2Δ/ent2Δ*, *ent2Δ/ent2Δ* plus *ENT2/ENT2,* or *ent2Δ/ent2Δ* plus *ENTH/ENTH* strains. All mice infected with *ent2Δ/ent2Δ* survived until the end of the experiment. Log-rank (Mantel-Cox) test; ***, *P < *0.001. Log-rank test for trend, *P = *0.0009. Eight mice were infected per group.

Morphogenesis is a key virulence trait, and as such, most mutants that cannot undergo this switch exhibit reduced virulence in a murine model of systemic infection (5). Therefore, we tested whether *ENT2* is required for virulence in a mouse model of systemic candidiasis. To do this, we utilized retro-orbital injection to introduce either wild type (CaSS1) or the *tetO-ENT2/ent2Δ* strain directly into the bloodstream of mice treated with either DOX or solvent in the drinking water to modulate expression of *ENT2* (Fig. 5A). While all mice infected with either the wild-type strain or the *tetO-ENT2*/*ent2Δ* mutant in the absence of DOX succumbed to infection by day 18, there was no mortality among DOX-treated animals infected with the *tetO-ENT2*/*ent2Δ* mutant, confirming that *ENT2* is required for C. albicans virulence in a mammalian host (*P* < 0.0001) (Fig. 5A). Thus, this work not only highlights the importance of Ent2 in governing hyphal morphogenesis *in vitro* but also reveals its key role in enabling virulence in a systemic model of infection.

To determine whether the entire Ent2 protein is required for virulence or whether virulence is mediated through the ENTH domain, we retro-orbitally infected mice with either wild-type (SN95), *ent2Δ/ent2Δ*, or *ent2Δ/ent2Δ* strains expressing two copies of either *ENT2* or just the ENTH domain reintroduced at the native locus. While all mice infected with either the wild-type, *ent2Δ/ent2Δ* plus *ENT2/ENT2*, or *ent2Δ/ent2Δ plus ENTH/ENTH* strains succumbed to infection by day 6, there was no mortality among animals infected with the *ent2Δ/ent2Δ* mutant, confirming that the ENTH domain is sufficient for C. albicans virulence in a mammalian host (*P* < 0.001) (Fig. 5B). Of note, the more rapid rate of death observed in this experiment than that depicted in Fig. 5A reflects a slightly higher inoculum (3 × 10^5^ versus 2 × 10^5^ CFU) and differences in the fungal strain background (SN95 versus GRACE reference strain).

Overall, these results support a model in which the ENTH domain of Ent2 binds Rga2 to regulate its localization and inhibit its interaction with Cdc42. This allows Cdc42 to remain active in response to filament-inducing cues and to signal to downstream effectors, including Cla4, resulting in filamentation (Fig. 4). When the ENTH-Rga2 interaction is abolished, Rga2 inhibits Cdc42 signaling, causing cells to proliferate as yeast (Fig. 4).

## DISCUSSION

In this study, we characterize the role of Ent2 in regulating C. albicans hyphal morphogenesis. Previous functional genomic studies had identified this protein as being important for hyphal morphogenesis in response to select inducing cues (15, 16, 21), but the mechanism by which Ent2 controls this key virulence trait remained elusive. Here, we expand on previous studies and find that genetic disruption of *ENT2* blocks filamentation under a wide range of liquid- and solid-inducing conditions and attenuates C. albicans virulence in a mouse model of systemic candidiasis. We discover that a single copy of Ent2’s ENTH domain is sufficient to regulate filamentous growth and virulence, and at least two ENTH domain residues, Y100 and T104, are required to mediate filamentation. Through coimmunoprecipitation experiments, we detected a physical interaction between the ENTH domain and the Cdc42 GAP, Rga2, with the ENTH residue Y100 being critical for this interaction, and we discovered this physical interaction to be required for proper Rga2 localization in yeast- and filament-inducing conditions. Further, genetic epistasis experiments revealed the interaction to be important for activating Cla4 signaling downstream of Cdc42 to govern hyphal morphogenesis. Overall, this work characterizes an essential regulator of filamentous growth to further elucidate the mechanisms underlying this key virulence trait in C. albicans.

Our discovery that Ent2 regulates filamentous growth in part through an interaction between its ENTH domain and Rga2 is consistent with previous findings that epsins mediate inhibition of RhoGAPs to promote GTPase-dependent signaling events important for cell polarity. In S. cerevisiae, the ENTH domain of either Ent1 or Ent2 is necessary and sufficient to sustain cell viability in an *ent1Δ ent2Δ* mutant (26). This essential function is mediated through key surface patch residues required for binding Cdc42 GAPs and, as such, mutating these residues results in defective Cdc42 activation (26). While C. albicans Ent2 is the only homolog of S. cerevisiae Ent1/2 and is not essential, our study highlights the importance of the ENTH domain in regulating RhoGAP localization and activity, which is conserved between the pathogen and model yeast. In mammalian cells, the ENTH domain is necessary and sufficient to rescue the impaired cell migration and invasion phenotypes that result from small interfering RNA (siRNA) knockdown of epsins (36). Specifically, mammalian epsins interact with the Cdc42/Rac1 GAP RalBP1 via their ENTH domain to enable proper activation of the RhoGTPase Rac1 and GTPase Arf6 (36). Taken together, these findings show that epsin regulation of GTPase signaling is conserved among diverse eukaryotes despite a divergence in cellular functions.

The results of our microscopy experiments suggest a mechanism whereby Ent2, through its ENTH domain, regulates Rga2 localization to ensure its proper function in both yeast and filaments. To our knowledge, this is the first instance where epsins have been shown to regulate localization of a RhoGAP in C. albicans. It is well established that in C. albicans, phosphorylation of Rga2 by Hgc1-Cdc28 inhibits Rga2 localization at the hyphal tip during filamentous growth (34), and this is in line with what we observed in ENTH^WT^ cells, as no foci were detected at the polarized tip (Fig. 3C). Interestingly, in S. cerevisiae, ENTH localizes to sites of Cdc42 activation (26), and it will be interesting in the future to determine how Ent2 is regulated in C. albicans and if it interacts with Cdc42 at the hyphal tip during filamentous growth.

We predicted that the role of epsins in filamentation is, at least in part, independent of their role in endocytosis, as the ubiquitin-interaction motifs, NPF tripeptides, and clathrin binding motif required for endocytosis reside in the C-terminal part of the protein outside the ENTH domain. However, we found that introduction of a single ENTH^WT^ domain was sufficient to restore normal endocytosis in the absence of Ent2 (Fig. 2D; see Fig. S4 in the supplemental material). This suggests that, in addition to its role in regulating Rga2, the ENTH domain plays a role in regulating efficient endocytosis. Notably, localization of epsins at sites of endocytosis may enhance their signaling function by specifically linking Cdc42 signaling to sites of polarized growth (26, 36). In line with this prediction, sites of endocytosis are polarized toward the growing hyphal tip during filamentous growth in C. albicans (5). The contribution of epsin localization to endocytic sites may explain our finding that the ENTH domain with mutations in residues predicted to be important for lipid binding (ENTH^R62L^ and ENTH^H72L^) did not fully restore filamentation to the same degree as ENTH^WT^ in the *ent2Δ/ent2Δ* background. Thus, these results are consistent with the model whereby epsins, via their ENTH domain, relieve GAP-mediated inhibition of GTPases to allow for signaling activation of cell polarity pathways, as well as to enable efficient endocytosis, which collectively contribute to filamentous growth.

We found that overexpression of Cla4 partially overcomes the requirement for the ENTH-Rga2 interaction to allow for filamentation. Failure to activate Cla4 in the absence of a functional ENTH domain is consistent with the observation that *cla4Δ/cla4Δ* mutants are blocked in filamentation under most inducing cues and are avirulent in mouse models of systemic candidiasis (33). Together with Cdc42, Cla4 mediates septin ring assembly at the hyphal tip, which is a key structure for establishing and maintaining polarized growth and inhibiting cell separation (5). Accordingly, in S. cerevisiae, the epsins are important for activating a Gic1-, Gic2-, and Bem1-dependent Cdc42 pathway, which is also implicated in regulating septin organization (26). Though Gic1 and Gic2 do not have homologues in C. albicans, it is possible that epsin-mediated regulation of septin structures has been conserved over evolutionary time despite a divergence in the genetic factors that are involved. Furthermore, studies from S. cerevisiae suggest that Bem1 acts as a scaffold to dock Cdc42, Cdc24, and Cla4 at sites of polarized growth (37). At these sites, Bem1 is proposed to act in a feedback loop with Cla4 to regulate phosphorylation of Cdc24, resulting in iterative rounds of Cdc42 GTPase cycling, which ultimately powers septin ring assembly (38). In accordance with this, S. cerevisiae Ent2 has been shown to physically interact with Cdc24 (39), and deletion of Ent2 affects Cdc24 localization (40), similar to what we observed with C. albicans ENTH and Rga2. Given that Cla4 overexpression only partially restored filamentation in the *ent2Δ/ent2Δ* plus ENTH^Y100R^ strain and that depletion of *RGA2* did not restore filamentation in the ENTH^Y100R^ background, it is likely that Ent2 regulates filamentous growth through Cdc42-dependent signaling, as well as endocytosis and other mechanisms that remain to be discovered.

These results unveil several exciting avenues of research to further elucidate how epsins regulate Cdc42 signaling and, specifically, how Ent2 regulates cellular processes involved in filamentation in C. albicans. We discovered that C. albicans Ent2 binds Rga2, but the potential for additional Ent2 targets remains. In S. cerevisiae, novel Ent2-specific activity was discovered when overexpression of the ENTH domain from Ent2, but not Ent1, resulted in significant cell division defects characterized by elongated, chained buds with aberrant septa (41). Results from this study indicated that Ent2 has a specific function in regulating cell division by physically binding the Cdc42 GAP, Bem3 (41). Interestingly, this ENTH2-specific function relies on a less-conserved cluster of residues (ScENTH2^N112, S114, E118^) that are different from what is found on ScENTH1 (ScENTH1^D112, E114, Q118^) and CaENTH (CaENTH^D112, T114, Q118^) (41). To further investigate how C. albicans Ent2 governs filamentous growth, it would be interesting to determine how Ent2 function is regulated and whether this regulation is different during yeast versus filamentous growth. Regardless, advancing our mechanistic insight into how regulators of hyphal morphogenesis influence this key virulence trait enables a deeper understanding of how this important pathogen causes disease. As human fungal pathogens such as C. albicans continue to pose a significant threat to human health, this knowledge has the potential to aid in the development of alternate antivirulence treatment strategies to help combat serious infections.

## MATERIALS AND METHODS

### Strains and culture conditions.

All strains, plasmids, and oligonucleotides used in this study are included in Tables S2 to S4 in the supplemental material, respectively. All C. albicans strains were archived in YPD medium (1% yeast extract, 2% peptone, 2% glucose) in 25% glycerol at −80°C. Strains were grown in YPD medium unless otherwise specified. For solid conditions, 2% agar was added. To repress gene expression from the *tetO* promoter in GRACE strains, strains were grown overnight in 1 μg/mL doxycycline (catalog no. DB0889; BioBasic; dissolved in water) and subcultured into the same conditions. We used 50 μg/mL doxycycline to repress *RGA2* expression in CaLC8185 and CaLC8186.

10.1128/mbio.03434-22.8 TABLE S2 Strains used in this study. Download Table S2, DOCX file, 0.02 MB. Copyright © 2023 Lash et al. 2023 Lash et al. https://creativecommons.org/licenses/by/4.0/ This content is distributed under the terms of the Creative Commons Attribution 4.0 International license.

### Filamentation assays.

For all liquid filamentation assays, saturated overnight cultures were diluted to an optical density at 600 nm (OD_600_) of 0.1 in the indicated media. Heat-inactivated newborn calf serum (catalog no. 26010074; Gibco) was used at 10% in RPMI 1640 medium with l-glutamine and sodium bicarbonate (catalog no. R8758; Sigma) with 5% CO_2_. For *N*-acetylglucosamine (GlcNAc), 5 mM was prepared in YNB medium (1× YNB, 2% Casamino Acids, 1× uridine, and 0.2% glucose). Spider medium and Lee’s medium were prepared as previously described (5). For all liquid media, strains were grown under shaking conditions. Filamentation was assessed at 5 h under all conditions. For solid cues, saturated overnight cultures were diluted 10-fold in water, and 5 μL was spotted onto the appropriate agar plates. Filamentation was assessed at 72 h. All strains were grown at 37°C except for the elevated temperature cue for which strains were grown in YPD medium at 39°C. Cell morphology for liquid conditions was captured using differential interference contrast (DIC) microscopy on a Zeiss Axio Imager.M1 (Carl Zeiss). Colony morphology for solid conditions was captured using a Bio-Rad ChemiDoc imaging system. Images are representative of two biological replicates.

### Fluorescence microscopy and endocytosis staining.

For GFP-Rga2 imaging, saturated overnight cultures were diluted to an OD_600_ of 0.1 in YPD medium and grown under shaking conditions at 30°C or 39°C for 4 h. Cells were washed with 1× phosphate-buffered saline (PBS) and imaged using DIC and fluorescence microscopy using a Zeiss Axio Imager.M1 at 40× with constant exposure. To assess endocytosis, saturated overnight cultures were diluted to an OD_600_ of 0.1 in YPD medium and grown under shaking conditions at 30°C for 4 h. We incubated 1 mL of cells with 8 μM FM4-64 (Thermo Fisher Scientific; catalog no. T3166) in the dark for 30 min at 30°C. Cells were washed with 1 mL YPD and resuspended in 4 mL YPD and incubated for 90 min at 30°C before being spun down, resuspended in 1× PBS, and visualized using the red fluorescent protein (RFP) filter on a Zeiss Axio Imager.M1 at 40× with constant exposure. A 0.7-OD unit of each cell culture was spun down and resuspended in a 100 μL YPD solution of 1 mg/mL Lucifer yellow (Invitrogen; catalog no. L682) and incubated at 30°C for 1 h. Cells were washed with 1× PBS and visualized using the GFP filter on a Zeiss Axio Imager.M1 at 40× with constant exposure.

### Quantitative reverse transcription-PCR.

Quantitative reverse transcription-PCR (qRT-PCR) was carried out as previously described (18). Briefly, saturated overnight cultures were subcultured to an OD_600_ of 0.1 in YPD medium and grown to mid-log phase before cell pellets were collected, flash frozen, and stored at −80°C. Cells were lysed by bead beating, and RNA was extracted and DNase treated using the RNAeasy kit (Qiagen) and DNA-free DNA removal kit (Invitrogen), respectively. cDNA was amplified using the iScript cDNA synthesis kit (Bio-Rad), and PCR was performed using the Fast SYBR green master mix (Applied Biosystems) and the Bio-Rad CFX-384 real-time system with the following cycling conditions: 95°C for 3 min, 95°C for 10 s, and 60°C for 30 s for 40 cycles. Reactions were performed in technical triplicate for two biological replicates, and data were analyzed using the Bio-Rad CFX Manager 3.1. All data were normalized to *ACT1* and *GPD1.* Error bars represent standard error of the mean.

### Protein alignment.

Amino acid sequences for S. cerevisiae *ENT1* (YDL161W), *ENT2* (YLR206W), and *RGA2* (YDR379W) and C. albicans *ENT2* (C2_01390W) and *RGA2* (C4_02000C) were retrieved from https://fungidb.org. Local alignment of amino acid sequences was performed using the Smith-Waterman algorithm (42). To be inclusive of all protein domain predictions from the InterPro family of databases on https://fungidb.org, the first 149 amino acids were used to define the ENTH domain.

### Western blotting.

Saturated overnight cultures of SN95, CaLC7767, CaLC7769, and CaLC7770 were subcultured to an OD_600_ of 0.1 in 50 mL YPD and incubated for 4 h at 30°C with shaking. Cells were pelleted at 3,000 rpm for 5 min at room temperature, washed with ice-cold water, pelleted again at 3,000 rpm for 5 min at 4°C, flash frozen, and stored at −80°C. Cell pellets were resuspended in 500 μL ice-cold lysis buffer (200 mM Tris [pH 7.5], 100 mM KCl, 5 mM MgCl_2_, 20% glycerol, 1× Roche protease inhibitor cocktail tablet [Roche Diagnostics], and 1 mM phenylmethylsulfonyl fluoride [PMSF; BioShop]) and lysed by bead beating twice for 4 min at 4°C, with 7 min on ice between cycles. Lysates were collected by stacked transfer and then clarified by centrifugation at 14,000 rpm for 20 min at 4°C. Protein levels were determined by a Bradford assay. Each sample was normalized to 2 mg/mL with lysis buffer, diluted into 1× sample buffer, and boiled for 5 min. We loaded 10 μg protein per sample on a 12% Tris-glycine SDS-PAGE gel. Separated proteins were transferred to polyvinylidene difluoride (PVDF) membrane (Bio-Rad) at 300 mA for 60 min. Blots were blocked with 5% skim milk in Tris-buffered saline with 0.1% Tween 20 (TBS-T). FLAG epitopes were detected using Monoclonal anti-FLAG M2-peroxidase (horseradish peroxidase [HRP]) antibody (1:3,000; Sigma; catalog no. A8592) in the block solution. Blots were washed with TBS-T before signals were detected using Clarity Western ECL substrate (Bio-Rad). Blots were performed in at least biological duplicate.

### Coimmunoprecipitation.

Saturated overnight cultures of SN95 (CaLC239), CaLC7767, and CaLC7769 were subcultured to an OD_600_ of 0.1 in 50 mL YPD and incubated for 4 h at 30°C or 39°C with shaking. Cells were pelleted at 3,000 rpm for 5 min at room temperature, washed with ice-cold water, pelleted again at 3,000 rpm for 5 min at 4°C, flash frozen, and stored at −80°C. Cell pellets were resuspended in 500 μL ice-cold lysis buffer (200 mM Na-HEPES [pH 7.5], 100 mM KCl, 20% glycerol, 1× Roche protease inhibitor cocktail tablet, and 1 mM PMSF) and lysed by bead beating twice for 4 min at 4°C, with 7 min on ice between cycles. Lysates were collected by stacked transfer and then clarified by centrifugation at 14,000 rpm for 20 min at 4°C. Protein levels were determined by a Bradford assay. For each reaction, 1 mL of lysate containing 2 mg total protein was incubated with 40 μL anti-FLAG M2 affinity agarose gel (Sigma) and rotated overnight at 4°C. The beads were washed five times with 1 mL ice-cold lysis buffer. The bound protein was eluted by boiling in 40 μL of 2× sample buffer. Input samples were normalized to 2 mg/mL with lysis buffer, diluted into 1× sample buffer, and boiled for 5 min. Ten micrograms of input samples and 25 μL of coimmunoprecipitation samples were loaded onto 12% Tris-glycine SDS-PAGE gels (for detection of ENTH-FLAG) and precast 10 well NuPage 3 to 8% Tris-acetate gels (Invitrogen; for detection of GFP-Rga2) to separate proteins. Separated proteins were transferred to polyvinylidene difluoride (PVDF) membranes at 300 mA for 60 min for Tris-glycine gels and 20 V for 60 min for precast gels. Blots were blocked with 5% skim milk in TBS-T. FLAG epitopes were detected using monoclonal anti-FLAG M2-peroxidase (HRP) antibody (1:3,000; Sigma; catalog no. A8592) in the block solution. GFP epitopes were detected using an anti-GFP antibody (1:3,000; Roche; catalog no. 11814460001) in block solution. To detect GFP, blots were washed with TBS-T and incubated with HRP-conjugated secondary antibody diluted 1:3,000 in block solution. Blots were washed with TBS-T before signals were detected using Clarity Western ECL substrate. Blots were performed in biological duplicate.

### X-ray crystallography.

Escherichia coli BL21(DE3) Gold was used for overexpression of CaEnt2 ENTH domain (residues 1 to 155) using the pMCSG68 vector and purified as described for CaAro1 (43). The CaEnt2 ENTH domain crystal was grown at room temperature using the vapor diffusion sitting drop method with a reservoir solution of 0.2 M NaCl, 0.1 M potassium citrate, pH 4.2, 20% (wt/vol) polyethylene glycol 8000 (PEG 8000), and 0.3 M NDSB-195. The crystal was cryoprotected using paratone oil. Diffraction data at 100 K was collected at a home source Rigaku HF-007 rotating anode R-Axis IV++ image plate detector. Diffraction data were processed using HKL3000 (44), and the structure was solved by molecular replacement (MR) using Phenix.phaser and the structure of the ScEnt2 ENTH domain (PDB accession no. 4GZC) (45). Model building and refinement were performed using Phenix.refine (46) and Coot (47). Atomic coordinates have been deposited in the Protein Data Bank with accession no. 5UCC.

### Strain construction.

**(i) CaLC7226.** Both alleles of *ENT2* were deleted using a transient CRISPR approach (23). The nourseothricin (NAT) deletion cassette with homology to *ENT2* was PCR amplified from pLC49 using oLC8581 and oLC8582. Cas9 was amplified from pLC963 using oLC6924 and oLC6925. Two fragments for the single guide RNA (sgRNA) were amplified from pLC963 with oLC5978 and oLC8579 (fragment A) and oLC8580 and oLC5980 (fragment B). Fusion PCR with nested primers oLC5981 and oLC5979 was performed on the two fragments to produce the sgRNA. All three pieces were then transformed into SN95. NAT-resistant transformants were selected for on YPD plates containing 150 μg/mL NAT. NAT-resistant colonies were PCR tested for upstream integration with oLC275 and oLC8583 and downstream integration with oLC274 and oLC8584. Homozygous deletion of *ENT2* was PCR verified by the lack of amplification of a wild-type allele using primers oLC8583 and oLC8585. This colony was grown in YNB-BSA (0.17% nitrogen base, 0.4% bovine serum albumin, 0.2% yeast extract, and 2% maltose) to induce expression of the FLP recombinase to excise the NAT marker.

**(ii) CaLC7766.** To truncate one copy of *ENT2* to just the ENTH domain (1 to 447 bp) and C-terminally tag with His_6_-FLAG_3_, the pLC1086 plasmid was amplified using oLC9746 (which has homology to the end of the ENTH domain and pLC1086) and oLC9747 (which has homology to the *ENT2* 3′ UTR and pLC1086). This piece was transformed into SN95. Histidine prototrophs were selected for on SD plates (6.7 g/L yeast nitrogen base, 2% glucose, and 1× arginine). Histidine prototrophs were PCR tested for upstream integration of the transformed DNA cassette with oLC8583 and oLC6915 and downstream integration of the transformed DNA cassette with oLC8584 and oLC6916.

**(iii) CaLC7767.** ENTH-His_6_-FLAG_3_-HIS was PCR amplified from CaLC7766 genomic DNA with primers oLC9688 and oLC8583. The correct 2,832-bp piece was gel purified and sent for sequencing with oLC9996 and oLC9683 and transformed into CaLC7226. Histidine prototrophs were selected for on synthetic defined (SD) plates lacking histidine. Histidine prototrophs were PCR tested for upstream integration of the transformed DNA cassette with oLC9682 and oLC6915 and downstream integration of the transformed DNA cassette with oLC6916 and oLC8584. Genomic DNA was extracted from a single transformant and was amplified with oLC9682 and oLC8584. The resulting PCR fragment was sent for Sanger sequencing with oLC9996 and oLC9683 to confirm no mutations were introduced into the ENTH domain.

**(iv) CaLC7769.** Overlapping forward primer oLC9752 and reverse primer oLC9753 containing the desired mutations (t298c_a299g) were paired either with reverse primer oLC8584 (3′ untranslated region [UTR]) or forward primer oLC9682 (5′ UTR) to amplify ENTH from CaLC7767 genomic DNA. These two pieces were fused using nested primers oLC9630 and oLC9688, and this piece was sent for Sanger sequencing with oLC9683 and oLC9996. The DNA cassette was then transformed into CaLC7226. Histidine prototrophs were selected for on SD plates lacking histidine. Histidine prototrophs were PCR tested for upstream integration of the transformed DNA cassette with oLC9682 and oLC6915 and downstream integration of the transformed DNA cassette with oLC6916 and oLC8584. Genomic DNA was extracted from a single transformant and was amplified with oLC9682 and oLC8584. The resulting PCR product was sent for Sanger sequencing with oLC9996 and oLC9683 to confirm only the desired mutations were introduced into the ENTH domain.

**(v) CaLC7770.** Overlapping forward primer oLC9754 and reverse primer oLC9755 containing the desired mutations (a310g_c311a) were paired either with reverse primer oLC8584 (3′ UTR) or forward primer oLC9682 (5′ UTR) to amplify ENTH from CaLC7767 genomic DNA. These two pieces were fused using nested primers oLC9630 and oLC9688, and this piece was sent for Sanger sequencing with oLC9683 and oLC9996. The DNA cassette was then transformed into CaLC7226. Histidine prototrophs were selected for on SD plates lacking histidine. Histidine prototrophs were PCR tested for upstream integration of the transformed DNA cassette with oLC9682 and oLC6915 and downstream integration of the transformed DNA cassette with oLC6916 and oLC8584. Genomic DNA was extracted from a single transformant and was amplified with oLC9682 and oLC8584. The resulting PCR product was sent for Sanger sequencing with oLC9996 and oLC9683 to confirm only the desired mutations were introduced into the ENTH domain.

**(vi) CaLC7772.** Overlapping forward primer oLC9758 and reverse primer oLC9759 containing the desired mutation (g185t) were paired either with reverse primer oLC8584 (3′ UTR) or forward primer oLC9682 (5′ UTR) to amplify ENTH from CaLC7767 genomic DNA. These two pieces were fused using nested primers oLC9630 and oLC9688, and this piece was sent for Sanger sequencing with oLC9683 and oLC9996. The DNA cassette was then transformed into CaLC7226. Histidine prototrophs were selected for on SD plates lacking histidine. Histidine prototrophs were PCR tested for upstream integration of the transformed DNA cassette with oLC9682 and oLC6915 and downstream integration of the transformed DNA cassette with oLC6916 and oLC8584. Genomic DNA was extracted from a single transformant and was amplified with oLC9682 and oLC8584. The resulting PCR product was sent for Sanger sequencing with oLC9996 and oLC9683 to confirm only the desired mutations were introduced into the ENTH domain.

**(vii) CaLC7773.** Overlapping forward primer oLC9760 and reverse primer oLC9761 containing the desired mutation (a215t) were paired either with reverse primer oLC8584 (3′ UTR) or forward primer oLC9682 (5′ UTR) to amplify ENTH from CaLC7767 genomic DNA. These two pieces were fused using nested primers oLC9630 and oLC9688, and this piece was sent for Sanger sequencing with oLC9683 and oLC9996. The DNA cassette was then transformed into CaLC7226. Histidine prototrophs were selected for on SD plates lacking histidine. Histidine prototrophs were PCR tested for upstream integration of the transformed DNA cassette with oLC9682 and oLC6915 and downstream integration of the transformed DNA cassette with oLC6916 and oLC8584. Genomic DNA was extracted from a single transformant and was amplified with oLC9682 and oLC8584. The resulting PCR product was sent for Sanger sequencing with oLC9996 and oLC9683 to confirm only the desired mutations were introduced into the ENTH domain.

**(viii) CaLC8185.** Both alleles of *RGA2* were promoter replaced with *tetO* and N-terminally tagged with GFP using a transient CRISPR approach (23). The *tetO-GFP* cassette with homology to the *RGA2* promoter was PCR amplified from pLC1470 using oLC10k71 and oLC10k72. Cas9 was amplified from pLC963 using oLC6924 and oLC6925. Two fragments for the sgRNA were amplified from pLC963 with oLC10k8 and oLC5978 (fragment A) and oLC10k7 and oLC5980 (fragment B). Fusion PCR with nested primers oLC5981 and oLC5979 was performed on the two fragments to produce the sgRNA. All three pieces were then transformed into CaLC7767. NAT-resistant transformants were selected for on YPD plates containing 150 μg/mL NAT. NAT-resistant colonies were PCR tested for upstream integration with oLC10k9 and oLC9723 and downstream integration with oLC10k10 and oLC9725. Tagging of both *RGA2* alleles was PCR verified by the lack of amplification of the wild-type promoter using primers oLC10k9 and oLC10k11. This colony was grown in YNB-BSA to induce expression of the FLP recombinase to excise the NAT marker.

**(ix) CaLC8186.** Both alleles of *RGA2* were promoter replaced with *tetO* and N-terminally tagged with GFP using a transient CRISPR approach (23). The *tetO-GFP* cassette with homology to the *RGA2* promoter was PCR amplified from pLC1470 using oLC10k71 and oLC10k72. Cas9 was amplified from pLC963 using oLC6924 and oLC6925. Two fragments for the sgRNA were amplified from pLC963 with oLC10k8 and oLC5978 (fragment A) and oLC10k7 and oLC5980 (fragment B). Fusion PCR with nested primers oLC5981 and oLC5979 was performed on the two fragments to produce the sgRNA. All three pieces were then transformed into CaLC7769. NAT-resistant transformants were selected for on YPD plates containing 150 μg/mL NAT. NAT-resistant colonies were PCR tested for upstream integration with oLC10k9 and oLC9723 and downstream integration with oLC10k10 and oLC9725. Tagging of both *RGA2* alleles was PCR verified by the lack of amplification of the wild-type promoter using primers oLC10k9 and oLC10k11. This colony was grown in YNB-BSA to induce expression of the FLP recombinase to excise the NAT marker.

**(x) CaLC8520.** The *TAR-tetO* cassette with homology to the *CLA4* promoter was PCR amplified from pLC605 using oLC10k384 and oLC10k385. Cas9 was amplified from pLC963 using oLC6924 and oLC6925. Two fragments for the sgRNA were amplified from pLC963 using oLC5978 and oLC10k383 (fragment A) and oLC5980 and oLC10k382 (fragment B). Fusion PCR with nested primers oLC5981 and oLC5979 was performed on the two fragments to produce the sgRNA. All three components were transformed into CaLC7769. NAT-resistant transformants were selected for on YPD plates containing 150 μg/mL NAT. NAT-resistant colonies were PCR tested for upstream integration with oLC9723 and oLC10k386 and downstream integration with oLC9729 and oLC10k387. Amplification of a band using primers targeting the wild-type promoter, oLC10k386 and oLC10k388, confirmed only one allele was promoter replaced. This colony was grown in YNB-BSA to induce expression of the FLP recombinase to excise the NAT marker.

**(xi) CaLC8521.** The *TAR-tetO* cassette with homology to the *CST20* promoter was PCR amplified from pLC605 using oLC10k377 and oLC10k378. Cas9 was amplified from pLC963 using oLC6924 and oLC6925. Two fragments for the sgRNA were amplified from pLC963 using oLC5978 and oLC10k376 (fragment A) and oLC5980 and oLC10k375 (fragment B). Fusion PCR with nested primers oLC5981 and oLC5979 was performed on the two fragments to produce the sgRNA. All three components were transformed into CaLC7769. NAT-resistant transformants were selected for on YPD plates containing 150 μg/mL NAT. NAT-resistant colonies were PCR tested for upstream integration with oLC9723 and oLC10k379 and downstream integration with oLC9729 and oLC10k380. Lack of amplification of a band using primers targeting the wild-type promoter, oLC10k379 and oLC10k381, confirmed both alleles were promoter replaced. This colony was grown in YNB-BSA to induce expression of the FLP recombinase to excise the NAT marker.

**(xii) CaLC8550.** Both copies of *ENT2* were reintroduced at the native locus in the *ent2Δ/Δ* genetic background using a transient CRISPR approach (23). Two fragments for the sgRNA were amplified from pLC963 with oLC10k657/oLC5980 (fragment A) and oLC10k658/oLC5978 (fragment B). Fusion PCR with nested primers oLC5981/oLC5979 was performed on the two fragments to produce the sgRNA targeting the FRT site. Cas9 was amplified from pC963 with oLC6924/oLC6925. The *ENT2* open reading frame was amplified from SN95 genomic DNA (gDNA) with primers oLC9630/oLC9631, the reverse primer having 20 bp of homology to the 5′ end of the arginine cassette (pLC45) to generate fragment A. To generate fragment B, the arginine cassette was amplified from pLC45 with oLC9632/oLC9633, with the forward primer containing homology of the end of the *ENT2* open reading frame (ORF) and homology to the 5′ end of the arginine cassette (pLC45) and the reverse primer containing homology to the 3′ end of the arginine cassette (pLC45) and the 3′ UTR of *ENT2.* Fusion PCR with nested primers oLC9636/oLC9637 was performed on the two fragments to generate the *ENT2-ARG* fusion construct. All three pieces were transformed into CaLC7226. Arginine prototrophs were selected for on SD plates lacking arginine. Arginine prototrophs were PCR tested for upstream integration of the transformed DNA cassette with oLC9683/oLC8585 and downstream integration with oLC9687/oLC8584. Integration of the DNA cassette at both loci was confirmed through PCR amplification of a 5,831-bp piece with oLC9682/oLC8584 and no amplification of a 1,266-bp piece spanning the FRT site.

**(xiii) CaLC8551.** Both copies of the *ENTH* domain were reintroduced at the native locus in the *ent2Δ/Δ* genetic background using a transient CRISPR approach (23). Two fragments for the sgRNA were amplified from pLC963 with oLC10k657/oLC5980 (fragment A) and oLC10k658/oLC5978 (fragment B). Fusion PCR with nested primers oLC5981/oLC5979 was performed on the two fragments to produce the sgRNA targeting the FRT site. Cas9 was amplified from pC963 with oLC6924/oLC6925. The *CaENT2 ENTH* domain (*ENT2*^1-444^) was amplified from SN95 gDNA with primers oLC9630/oLC9634, with the reverse primer having 20 bp of homology to the 5′ end of the arginine cassette (pLC45) to generate fragment A. To generate fragment B, the arginine cassette was amplified from pLC45 with oLC9635/oLC9633, with the forward primer containing homology to the end of the ENTH domain and homology to the 5′ end of the arginine cassette (pLC45) and the reverse primer containing homology to the 3′ end of the arginine cassette (pLC45) and the 3′ UTR of *ENT2*. Fusion PCR with nested primers oLC9636/oLC9637 was performed on the two fragments to generate the *ENTH-ARG* fusion construct. All three pieces were transformed into CaLC7226. Arginine prototrophs were selected for on SD plates lacking arginine. Arginine prototrophs were PCR tested for upstream integration of the transformed DNA cassette with oLC9682/oLC8585 and downstream integration with oLC9687/oLC8584. Integration of the DNA cassette at both loci was confirmed through PCR amplification of a 5,394-bp piece with oLC9682/oLC8584 and no amplification of a 1,266-bp piece spanning the FRT site.

**(xiv) CaLC8552.** The NEUT5L::His3_6_FLAG_3_-ARG cassette was PCR amplified from pLC1085 by oLC9008/oLC9009 and transformed into CaLC7226. Arginine prototrophs were selected for on SD plates lacking arginine. Arginine prototrophs were PCR tested with oLC6729/oLC6915 for upstream integration and oLC6916/oLC6730 for downstream integration.

**(xv) CaLC8553.** The NEUT5L::His3_6_FLAG_3_-ARG cassette was PCR amplified from pLC1085 by oLC9008/oLC9009 and transformed into SN95. Arginine prototrophs were selected for on SD plates lacking arginine. Arginine prototrophs were PCR tested with oLC6729/oLC6915 for upstream integration and oLC6916/oLC6730 for downstream integration.

### Systemic infection model.

Animal experiments were conducted with approval from UCSF Institutional Animal Care and Use Committee (protocol number AN189431-01). The survival curve was performed with 8- to 10-week-old BALB/c (no. 028) mice from Charles River Laboratories. For infections with GRACE strains, mice were separated into two groups, animals without treatment and animals treated with 0.25 mg/mL of DOX and 5% glucose via drinking water beginning 7 days prior to infection and continued throughout the experiment. Strains were grown in yeast extract-peptone-dextrose (YEPD) with or without DOX overnight at 30°C and diluted to an OD_600_ of 0.1 in 100 mL YEPD broth with or without DOX and incubated at 30°C with constant shaking. Systemic infection was performed by inoculation of 2 × 10^5^ CFU of mid-log-phase cells into the retro bulbar sinus of mice under isoflurane anesthesia. For non-GRACE strains, strains were grown in YEPD overnight at 30°C and diluted to an OD_600_ of 0.1 in 100 mL YEPD broth and incubated at 30°C with constant shaking. Systemic infection was performed by inoculation of 3 × 10^5^ CFU of mid-log-phase cells into the retrobulbar sinus of mice under isoflurane anesthesia. Mice were monitored closely and euthanized upon development of signs of clinical morbidity (defined as body condition score of ≤2, hunched posture, and decreased motor activity).

10.1128/mbio.03434-22.9 TABLE S3 Plasmids used in this study. Download Table S3, DOCX file, 0.02 MB. Copyright © 2023 Lash et al. 2023 Lash et al. https://creativecommons.org/licenses/by/4.0/ This content is distributed under the terms of the Creative Commons Attribution 4.0 International license.

10.1128/mbio.03434-22.10 TABLE S4 Primers used in this study. Download Table S4, DOCX file, 0.03 MB. Copyright © 2023 Lash et al. 2023 Lash et al. https://creativecommons.org/licenses/by/4.0/ This content is distributed under the terms of the Creative Commons Attribution 4.0 International license.
