# Supplementary figures and images for: Ent2 Governs Morphogenesis and Virulence in Part through Regulation of the Cdc42 Signaling Cascade in the Fungal Pathogen Candida albicans
Source: mBio. 2023 Feb 21;14(2):e03434-22. doi: 10.1128/mbio.03434-22 (PMC10128014; doi:10.1128/mbio.03434-22)

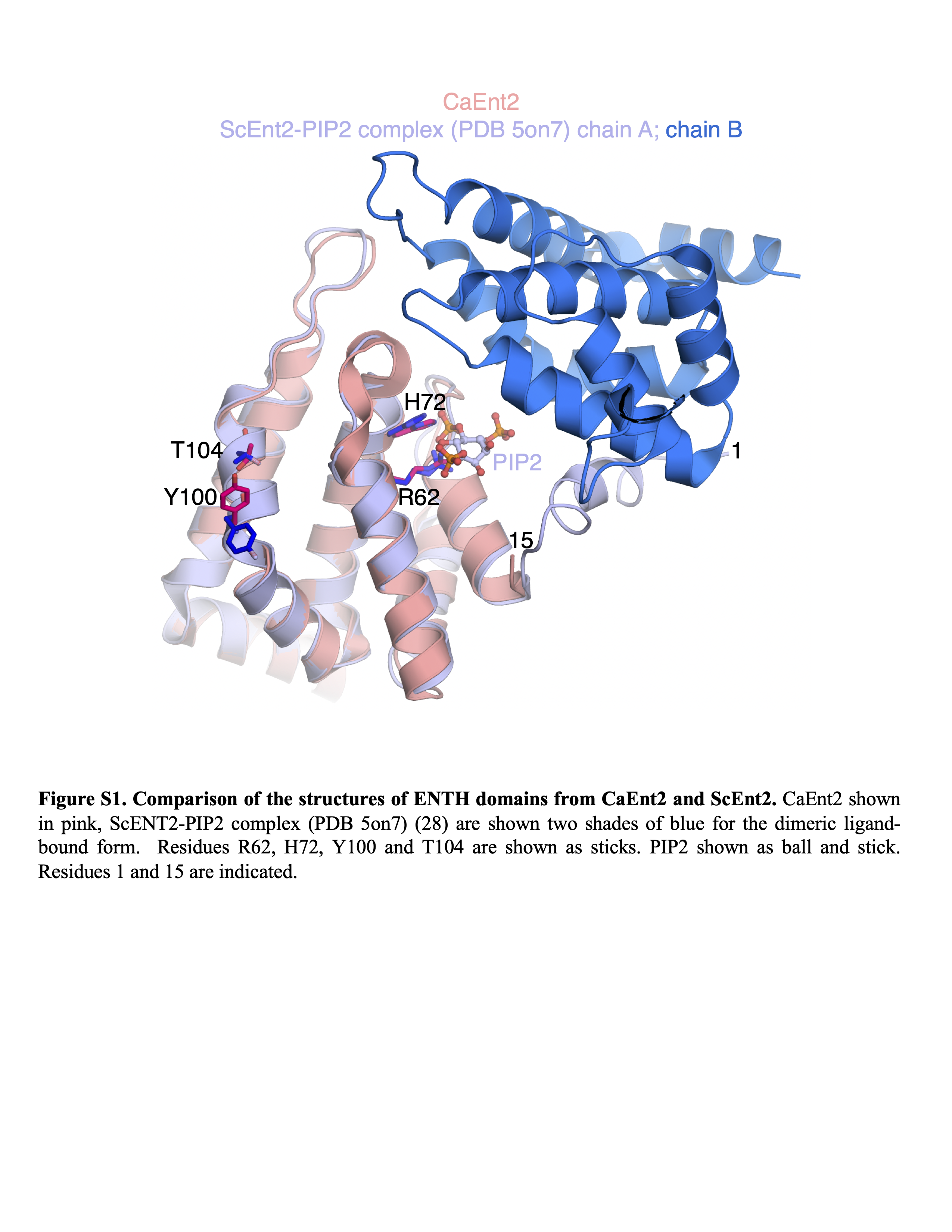

Supplement: FIG S1 [file mbio.03434-22-s0001.tif]

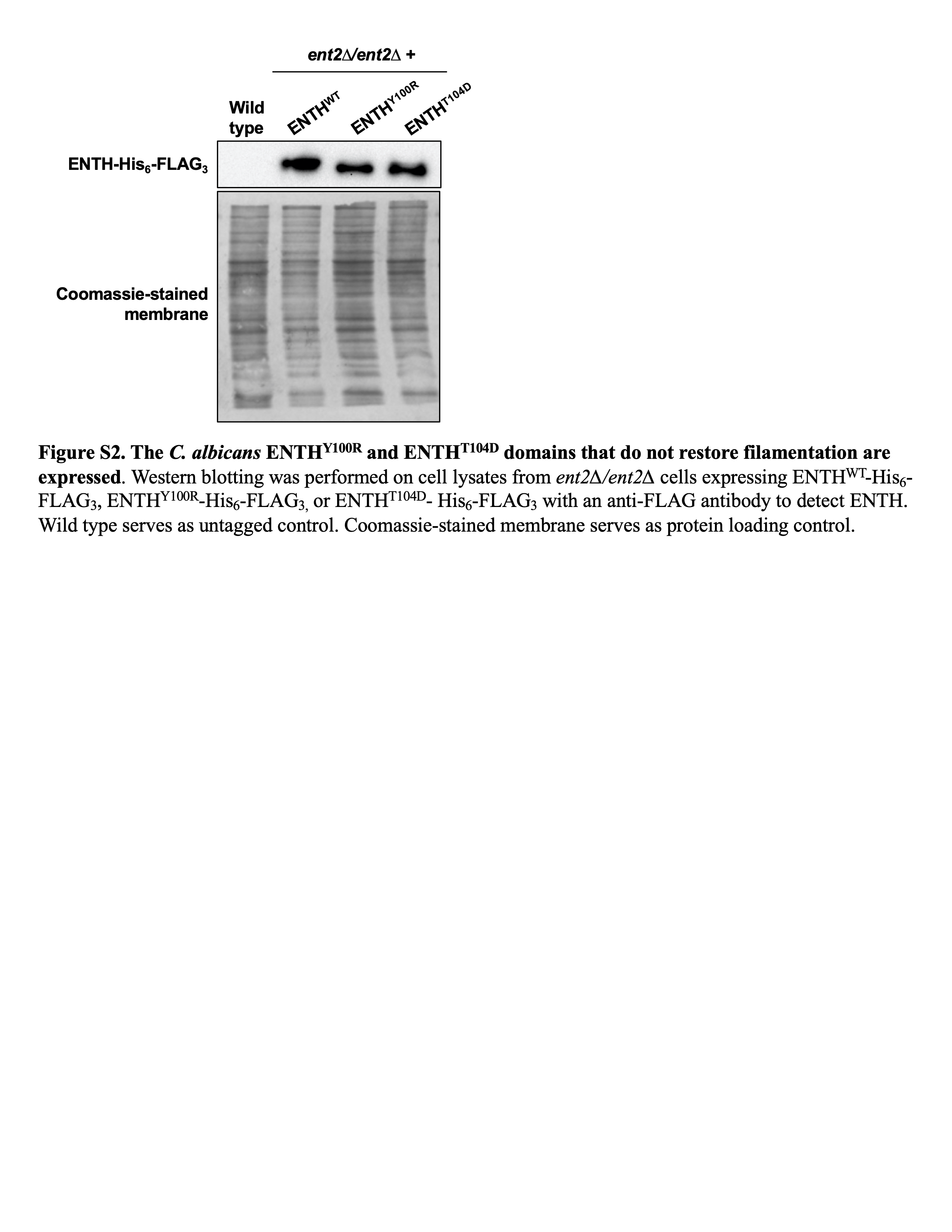

Supplement: FIG S2 [file mbio.03434-22-s0002.tif]

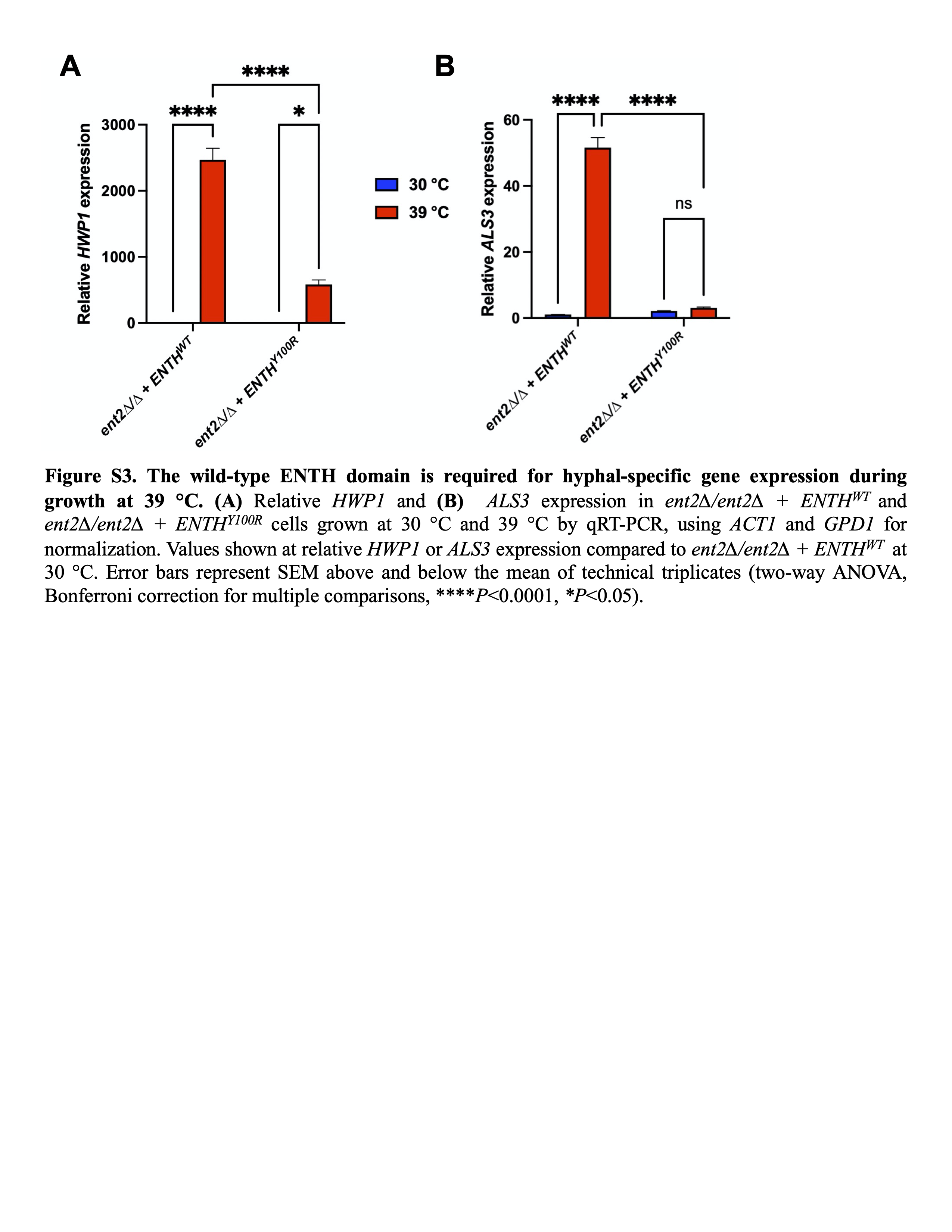

Supplement: FIG S3 [file mbio.03434-22-s0003.tif]

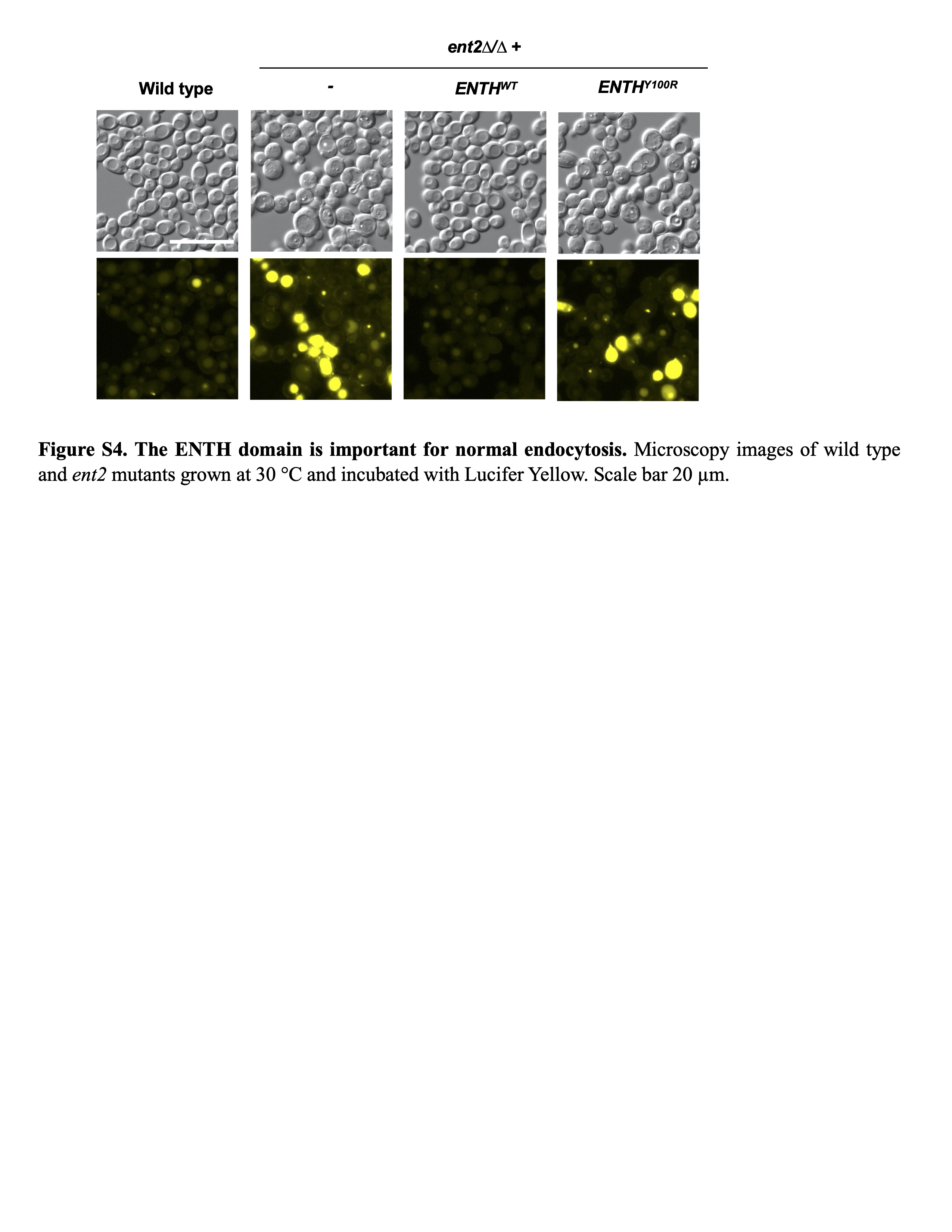

Supplement: FIG S4 [file mbio.03434-22-s0004.tif]

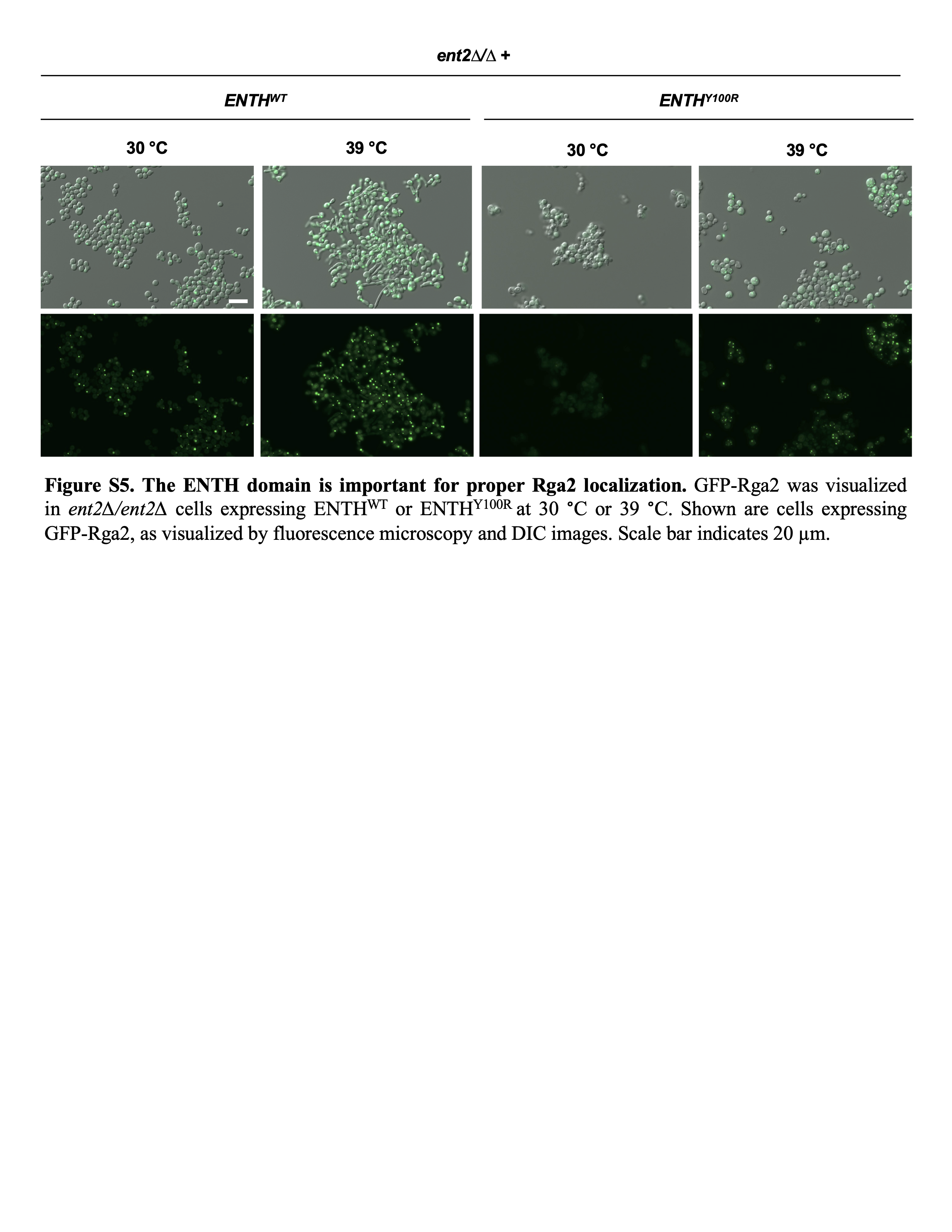

Supplement: FIG S5 [file mbio.03434-22-s0005.tif]

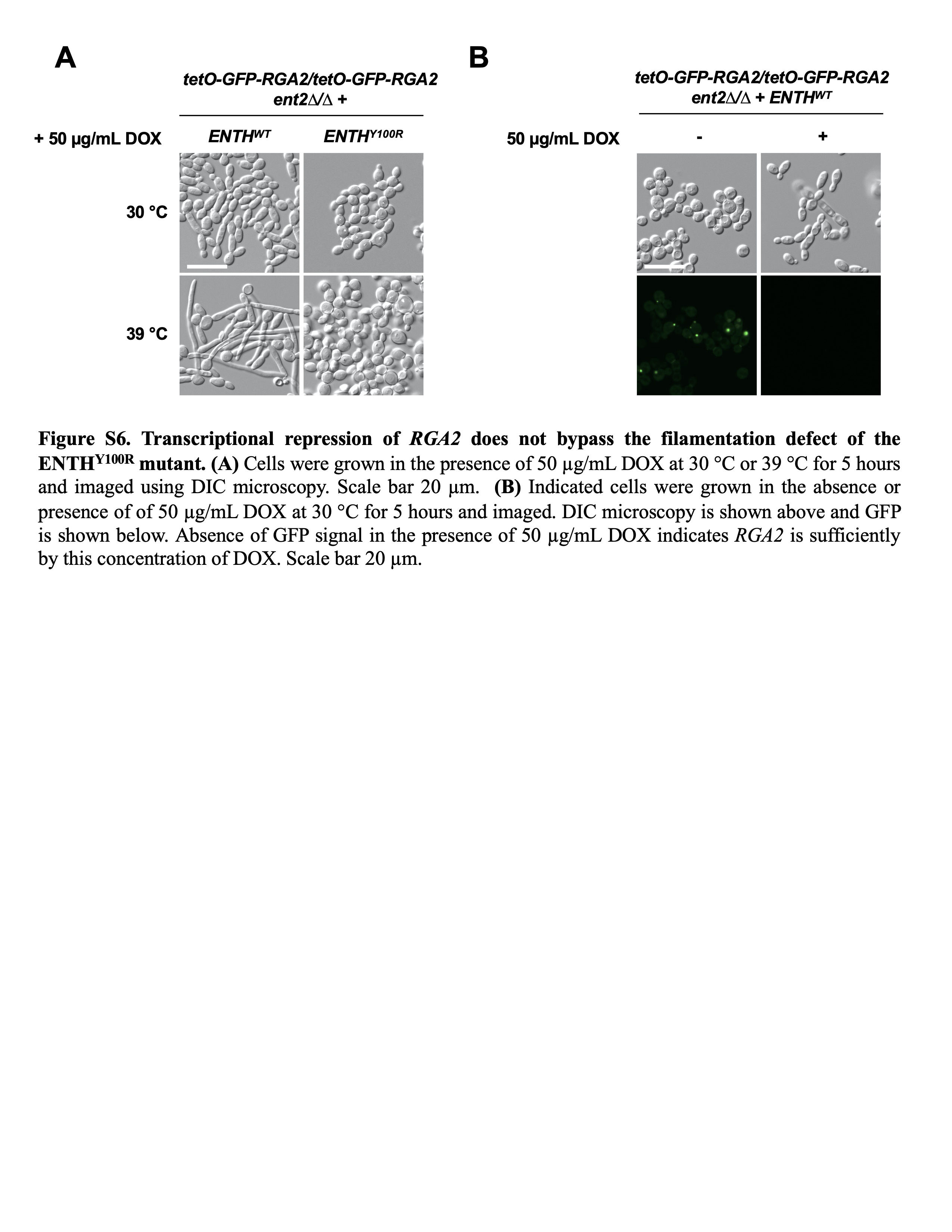

Supplement: FIG S6 [file mbio.03434-22-s0006.tif]
