# Supplementary material for: Ent2 Governs Morphogenesis and Virulence in Part through Regulation of the Cdc42 Signaling Cascade in the Fungal Pathogen Candida albicans
Source: mBio. 2023 Feb 21;14(2):e03434-22. doi: 10.1128/mbio.03434-22 (PMC10128014; doi:10.1128/mbio.03434-22)
Supplement: TABLE S1 [file mbio.03434-22-s0007.docx]

**Table S1. X-ray crystallographic statistics.**

| *Data collection* |  |
| --- | --- |
| Space group | H 3 |
| Unit cell  a, b, c (Å)  α, β, γ, (°) | 88.90, 88.90, 67.26  90, 90, 120 |
| Resolution, Å | 25.0 – 1.83 |
| R_merge_^a^  R_pim_^b^ | 0.066 (1.185)^*^  0.028 (0.527) |
| CC_1/2_^*^ | 0.522 |
| *I*/σ (*I*) | 41.73 (2.04) |
| Completeness, % | 99.3 (97.6) |
| Redundancy | 6.5 (5.7) |
| *Refinement* |  |
| Resolution, Å | 22.42 – 1.83 |
| No. unique reflections:  working, test | 17420, 863 |
| R-factor/free R­-factor^c^ | 15.8/18.5 (27.4/33.1) |
| No. refined atoms  Protein  Solvent  Water | 1134  30  217 |
| *B*-factors  Protein  Solvent  Water | 37.2  50.61  57.9 |
| r.m.s.d.  Bond lengths, Å  Bond angles, ° | 0.008  0.947 |

*All values in brackets and CC_1/2_ values refer to highest resolution shells.

^a^*R*_merge_ = Σ_hkl_Σ_j_|*I*_hkl.j_ - ​​〈*I*_hkl_〉|/Σ_hkl_Σ_j_*I*_hk,j_, where *I*_hkl,j_ and〈*I*_hk,j_〉are the *j*th and mean measurement of the intensity of reflection *j*.

^b^*R*_pim_ = Σ_hkl_√(n/n-1) Σ^n^_j=1_|*I*_hkl.j_ - 〈*I*_hkl_〉|/Σ_hkl_Σ_j_I_hk,j_

^c^*R* = Σ|F_p_^obs^ – F_p_^calc^|/ΣF_p_^obs^, where F_p_^obs^ and F_p_^calc^ are the observed and calculated structure factor amplitudes, respectively.
