# Supplementary material for: Ent2 Governs Morphogenesis and Virulence in Part through Regulation of the Cdc42 Signaling Cascade in the Fungal Pathogen Candida albicans
Source: mBio. 2023 Feb 21;14(2):e03434-22. doi: 10.1128/mbio.03434-22 (PMC10128014; doi:10.1128/mbio.03434-22)
Supplement: TABLE S2 [file mbio.03434-22-s0008.docx]

**Supplementary Table 2: Strains used in this study.**

| **Strain** | **Alias** | **Genotype** | **Source** |
| --- | --- | --- | --- |
| CaLC239 | SN95 | *arg/arg4 his1/his1 URA3/ura3::imm^434^ IRO1/iro1::imm^434^* | (1) |
| CaLC6106 | GRACE parent strain (CaSS1) | *ura3::imm^434^/ura3::imm^434^ his3::hisG/his3::hisG leu2::tetR- GAL4AD-URA/LEU2* | (2) |
| GRACE strain | *tetO-ENT2/ent2∆* | As GRACE parent *SAT1::tetO-ENT2/ent2::HIS3* | (2) |
| CaLC7226 | SN95 *ent2∆/ent2∆* | *arg/arg4 his1/his1 URA3/ura3::imm^434^ IRO1/iro1::imm^434^ ent2::FRT/ent2::FRT* | This study |
| CaLC7766 | SN95 *ENTH^WT^/ENT2* | *arg/arg4 his1/his1 URA3/ura3::imm434 IRO1/iro1::imm434 ent2(1-447)-6his3Flag-HIS/ENT2* | This study |
| CaLC7767 | SN95 ENTH^WT^-*6his3flag/ent2∆* | *arg/arg4 his1/his1 URA3/ura3::imm^434^ IRO1/iro1::imm^434^ ent2::ent2(1-447)-6his3Flag-HIS/ent2::FRT* | This study |
| CaLC7769 | SN95 ENTH^Y100R^-*6his3flag/ent2∆* | *arg/arg4 his1/his1 URA3/ura3::imm^434^ IRO1/iro1::imm^434^ ent2::ent2(1-447)^T298C, A299G^-6his3Flag-HIS/ent2::FRT* | This study |
| CaLC7770 | SN95 ENTH^T104D^-*6his3flag/ent2∆* | *arg/arg4 his1/his1 URA3/ura3::imm^434^ IRO1/iro1::imm^434^ ent2::ent2(1-447)^A310G, C311A^-6his3Flag-HIS/ent2::FRT* | This study |
| CaLC7772 | SN95 ENTH^R62L^-*6his3flag/ent2∆* | *arg/arg4 his1/his1 URA3/ura3::imm^434^ IRO1/iro1::imm^434^ ent2::ent2(1-447)^G185T^-6his3Flag-HIS/ent2::FRT* | This study |
| CaLC7773 | SN95 ENTH^H72L^-*6his3flag/ent2∆* | *arg/arg4 his1/his1 URA3/ura3::imm^434^ IRO1/iro1::imm^434^ ent2::ent2(1-447)^A215T^-6his3Flag-HIS/ent2::FRT* | This study |
| CaLC8185 | SN95 ENTH^WT^-*6his3flag/ent2∆* TAR-tetO-GFP-RGA2/TAR-tetO-GFP-RGA2 | *arg/arg4 his1/his1 URA3/ura3::imm^434^ IRO1/iro1::imm^434^ ent2::ent2(1-447)-6his3Flag-HIS/ent2::FRT TAR-tetO-GFP-RGA2/TAR-tetO-GFP-RGA2* | This study |
| CaLC8186 | SN95 ENTH^Y100R^-*6his3flag/ent2∆* TAR-tetO-GFP-RGA2/TAR-tetO-GFP-RGA2 | *arg/arg4 his1/his1 URA3/ura3::imm^434^ IRO1/iro1::imm^434^ ent2::ent2(1-447)^T298C, A299G^-6his3Flag-HIS/ent2::FRT TAR-tetO-GFP-RGA2/TAR-tetO-GFP-RGA2* | This study |
| CaLC8520 | SN95 ENTH^Y100R^-*6his3flag/ent2∆* TAR-tetO-CLA4/CLA4 | *arg/arg4 his1/his1 URA3/ura3::imm^434^ IRO1/iro1::imm^434^ ent2::ent2(1-447)^T298C, A299G^-6his3Flag-HIS/ent2::FRT TAR-tetO-CLA4/CLA4* | This study |
| CaLC8521 | SN95 ENTH^Y100R^-*6his3flag/ent2∆* TAR-tetO-CST20/TAR-tetO-CST20 | *arg/arg4 his1/his1 URA3/ura3::imm^434^ IRO1/iro1::imm^434^ ent2::ent2(1-447)^T298C, A299G^-6his3Flag-HIS/ent2::FRT TAR-tetO-CST20/TAR-tetO-CST20* | This study |
| CaLC8550 | CaLC7226 + *ENT2/ENT2* | *arg/arg4 his1/his1 URA3/ura3::imm^434^ IRO1/iro1::imm^434^ ENT2-ARG/ENT2-ARG* | This study |
| CaLC8551 | CaLC7226 + *ENTH/ENTH* | *arg/arg4 his1/his1 URA3/ura3::imm^434^ IRO1/iro1::imm^434^ ENTH-ARG/ENTH-ARG* | This study |
| CaLC8225 | CaLC7226 + NEUT5L::6his3flag-ARG | *arg4/arg4 his1/his1 URA3/ura3::imm434 IRO1/iro1::imm434 NEUT5L::6his3flag-ARG/NEUT5L ent2::FRT/ent2::FRT* | This study |
| CaLC8553 | SN95 + NEUT5L::6his3flag-ARG | *arg4/arg4 his1/his1 URA3/ura3::imm434 IRO1/iro1::imm434 NEUT5L::6his3flag-ARG/NEUT5L* | This study |

1. Noble SM, Johnson AD. 2005. Strains and strategies for large-scale gene deletion studies of the diploid human fungal pathogen *Candida albicans*. *Eukaryot Cell* 4:298–309.

2. Roemer T, Jiang B, Davison J, Ketela T, Veillette K, Breton A, Tandia F, Linteau A, Sillaots S, Marta C, Martel N, Veronneau S, Lemieux S, Kauffman S, Becker J, Storms R, Boone C, Bussey H. 2003. Large-scale essential gene identification in *Candida albicans* and applications to antifungal drug discovery. *Mol Microbiol* 50:167–181.
