# Supplementary material for: Ent2 Governs Morphogenesis and Virulence in Part through Regulation of the Cdc42 Signaling Cascade in the Fungal Pathogen Candida albicans
Source: mBio. 2023 Feb 21;14(2):e03434-22. doi: 10.1128/mbio.03434-22 (PMC10128014; doi:10.1128/mbio.03434-22)
Supplement: TABLE S3 [file mbio.03434-22-s0009.docx]

**Supplementary Table 3: Plasmids used in this study.**

| **Plasmid ID** | **Description** | **Source** |
| --- | --- | --- |
| pLC49 | p863 (for gene disruption with *FLP-NAT*) | (2) |
| pLC963 | pV1393-1 (CaCas9/sgRNA entry expression vector, contains NatR gene, targeting *NEUT5L* | (3) |
| pLC1086 | pFA-6his3Flag-HIS | (4) |
| pLC1470 | pLC605-GFP | (5) |
| pLC605 | *CaTAR-FLP-NAT-tetO* | (3) |
| pLC45 | pSN69 (for gene disruption with CdARG4) | (1) |
| pLC1085 | pFA-6his3Flag-ARG | (4) |

1. Noble SM, Johnson AD. 2005. Strains and strategies for large-scale gene deletion studies of the diploid human fungal pathogen *Candida albicans*. *Eukaryot Cell* 4:298–309.

2. Shen J, Guo W, Köhler JR. 2005. CaNAT1, a heterologous dominant selectable marker for transformation of *Candida albicans* and other pathogenic *Candida* species. *Infect Immun* 73:1239–1242.

3. Veri AO, Miao Z, Shapiro RS, Tebbji F, O’Meara TR, Kim SH, Colazo J, Tan K, Vyas VK, Whiteway M, Robbins N, Wong KH, Cowen LE. 2018. Tuning Hsf1 levels drives distinct fungal morphogenetic programs with depletion impairing Hsp90 function and overexpression expanding the target space. *PLoS Genet* 14:e1007270.

4. Zhang A, Petrov KO, Hyun ER, Liu Z, Gerber SA, Myers LC. 2012. The Tlo proteins are stoichiometric components of *Candida albicans* mediator anchored via the Med3 subunit. *Eukaryot Cell* 11:874–884.

5. Revie NM, Iyer KR, Maxson ME, Zhang J, Yan S, Fernandes CM, Meyer KJ, Chen X, Skulska I, Fogal M, Sanchez H, Hossain S, Li S, Yashiroda Y, Hirano H, Yoshida M, Osada H, Boone C, Shapiro RS, Andes DR, Wright GD, Nodwell JR, Del Poeta M, Burke MD, Whitesell L, Robbins N, Cowen LE. 2022. Targeting fungal membrane homeostasis with imidazopyrazoindoles impairs azole resistance and biofilm formation. *Nat Commun* 13:3634.
