# Supplementary material for: Ent2 Governs Morphogenesis and Virulence in Part through Regulation of the Cdc42 Signaling Cascade in the Fungal Pathogen Candida albicans
Source: mBio. 2023 Feb 21;14(2):e03434-22. doi: 10.1128/mbio.03434-22 (PMC10128014; doi:10.1128/mbio.03434-22)
Supplement: TABLE S4 [file mbio.03434-22-s0010.docx]

**Supplementary Table 4: Primers used in this study.**

| Oligo | Description | Sequence (5' to 3') |
| --- | --- | --- |
| oLC2285 | *CaACT1AB+855-F* | GACCTTGAGATACCCAATTG |
| oLC2286 | *CaACT1AB+1076-R* | CAGCTTGAATGGAAACGTAG |
| oLC752 | *GPD1A+570-F* | AGTATGTGGAGCTTTACTGGGA |
| oLC753 | *GPD1AB+766-R* | CAGAAACACCAGCAACATCTTC |
| oLC10k570 | *CaCLA4 + 437 R* | TTGCTATACCCGGAGTTTGG |
| oLC4706 | *CaCLA4 + 237 F* | GCAAACGTCAATCAGGTTGG |
| oLC10k568 | *CaCST20 + 276 F* | CTGGGGAAACTAATGATAGG |
| oLC10k569 | *CaCST20 + 491 R* | TTAGTTAGCTCGGTGTACTG |
| oLC9996 | *CaENTH+95F* | ACCCACCACGTTTGATATGG |
| oLC8585 | *CaENT22+249-R* | TTGTCCGATCCATAACGAAC |
| oLC10k10 | *CaRga2-428dn-R* | TCTTGGCCATTAGTTTCTGG |
| oLC10k11 | *CaRga2-88up-R* | GTGTAGGGTATAATCAAGCG |
| oLC10k375 | *CaCst20_sgRNA-F* | CCAAGTGCTATAACTCACAAGTTTTAGAGCTAGAAATAGCAAGTTAAAA |
| oLC10k376 | *CaCst20_sgRNA-R* | TTGTGAGTTATAGCACTTGGCAAATTAAAAATAGTTTACGCAAGTC |
| oLC10k377 | *CaCst20_pLC605-F* | AATTACTAATAGTTTTCAATTAATATTCACAATTTAACTATTTGTTTGACAGCTGAAAAGAGATAAAAAAGGAAACAGCTATGACCATG |
| oLC10k378 | *CaCst20_pLC605-R* | GTAGATGAGAAGACTCATTTGGATCTGTTATTGATGTTTGTGTAGGATTGTTCTCTGAAAGTATGCTCATCGACTATTTATATTTGTATG |
| oLC10k379 | *CaCst20_up593-F* | TAGTAGTTTGTTAGTAGCGG |
| oLC10k380 | *CaCst20_dn181-R* | CTGATGTAGTATTCATGACC |
| oLC10k381 | *CaCst20_up40-R* | AGTTAAATTCAATGGGGAGG |
| oLC10k382 | *CaCla4_sgRNA-F* | GTTGAAAAAAAAAAAATAGGGTTTTAGAGCTAGAAATAGCAAGTTAAAA |
| oLC10k383 | *CaCla4_sgRNA-R* | CCTATTTTTTTTTTTTCAACCAAATTAAAAATAGTTTACGCAAGTC |
| oLC10k384 | *CaCla4_pLC605-F* | GTCAGGGATAGTTTAGTAAATATTTACATTCTCCATTCAATCCTAAATTTTTTTTTATATAGCTAGTTTTGGAAACAGCTATGACCATG |
| oLC10k385 | *CaCla4_pLC605-R* | CAGCTGCCCCATTTGGTGGAGGTGGCGCACGTCTATGGTTTTTCAAATCTGATGTATAAATACTTGTCATCGACTATTTATATTTGTATG |
| oLC10k386 | *CaCla4_up547-F* | ATATTATTGTCTTCCTCCTC |
| oLC10k387 | *CaCla4_dn191-R* | TTGGTTTGATTTGCTGTTAC |
| oLC10k388 | *CaCla4_up77-R* | GGTAATATAACTGACAAGAC |
| oLC10k7 | *CaRga2-sgRNA-F* | ATATCATTCAAATAACCAGTGTTTTAGAGCTAGAAATAGCAAGTTAAAA |
| oLC10k71 | *CaRga2-tetOGFP-F* | AATTCAACGGAAAATATAAGAAAAAGTTTTAAATTTAAGTTTTTGGTCTTTTTTTTTGCCCGCAAACTGAGGAAACAGCTATGACCATG |
| oLC10k72 | *CaRga2-tetOGFP-R* | AAGGTTGCGAGGATAACTCGGGTGTCTCATGTATATGGGGCCGATTGTTTGTTGGTGGCACAAGTTCAGGACCTCCCTGCGGTGACTT |
| oLC10k8 | *CaRga2-sgRNA-R* | ACTGGTTATTTGAATGATATCAAATTAAAAATAGTTTACGCAAGTC |
| oLC10k9 | *CaRga2-481up-F* | TTTTGGTGATTTCCCTAAGG |
| oLC274 | *pJK863down-F* | CTGTCAAGGAGGGTATTCTGG |
| oLC275 | *pJK863up-R* | AAAGTCAAAGTTCCAAGGGG |
| oLC5978 | *pLC963-SNR52-F* | GACTGTCAAGGAGGGTATTC |
| oLC5979 | *pLC963-SNR52-N-F* | CCGCAAGTGATTAGACTTAG |
| oLC5980 | *pLC963-sgRNA-R* | GAATACCACTTGTTTACCGG |
| oLC5981 | *pLC963-sgRNA-N-R* | GGTGGCGGCAAAACTAATTC |
| oLC6915 | *pLC516 R (Kpp067)* | TAACTTCTGTCTCCTCATCCTC |
| oLC6916 | *pLC1869 F (Kpp050)* | TTTAAAGTCAATAGGCATTCTCG |
| oLC6924 | *CaCas9/for* | ATCTCATTAGATTTGGAACTTGTGGGTT |
| oLC6925 | *CaCas9/rev* | TTCGAGCGTCCCAAAACCTTCT |
| oLC8579 | *CaEnt2_sgRNA+212-R* | TCAATGATTTGGCCACATGTCAAATTAAAAATAGTTTACGCAAGTC |
| oLC8580 | *CaEnt2_sgRNA+212-F* | ACATGTGGCCAAATCATTGAGTTTTAGAGCTAGAAATAGCAAGTTAAAA |
| oLC8581 | *CaEnt2_pLC49-F* | ACTAGACAAAGTCTTCACCCTCCCCCCAAATAAAAAGGGTTTATATCATTATATACGGATTTTTGAAATTGGAAACAGCTATGACCATG |
| oLC8582 | *CaEnt2_pLC49-R* | TGTTGTTGTTGTTATATGCTATCAATATAACATCAATTCTAAAAAGCAATTATTGTTGATTGGAACTTGTGTAAAACGACGGCCAG |
| oLC8583 | *CaEnt2+184-F* | TTGTTGTCGTTGTTGCTGAG |
| oLC8584 | *CaEnt2+2254-R* | TCAAAGGGGGATACCGAAAC |
| oLC8585 | *CaEnt2+249-R* | TTGTCCGATCCATAACGAAC |
| oLC9630 | *CaEnt2_P1-F* | ATCGGAATTCGTGCGAAACC |
| oLC9682 | *CaEnt2-345-F* | TATATGGCGTAAGTTAGACC |
| oLC9683 | *CaEnt2+445-R* | TCTTTTTAGCATTAGCACGC |
| oLC9688 | *CaEnt2+1870-R* | TTTAGAATGGGATTGGTGTG |
| oLC9723 | *TAR-rev* | CCCAACAATGCCAATACTCC |
| oLC9725 | *GFP-fwd* | GGCTGACAAACAAAAGAATGG |
| oLC9729 | *tetO-fwd* | CATGTTTGTCGTTTCTGATGG |
| oLC9746 | *CaENTH-pLC1086-F* | TATTAGAGTTAAAGCTAAGGAATTGGTATCACTTTTGAGAGACGATGAGCGATTAAAACAAGAGCGTGCTAATGCTAAAAAGAATGGTCGACGGATCCCC |
| oLC9747 | *CaENTH-pLC1086-R* | ATACATAGTTGTTGTTGTTGTTGTTATATGCTATCAATATAACATCAATTCTAAAAAGCAATTATTGTTGATTGGAACTTGTTCGATGAATTCGAGCTCG |
| oLC9752 | *CaENTH Y100RF* | ATGTGTGTTATGGGCCAAAGACAATCTTCGCATTATTAAAACTTTAAGAGAATTTGTTC |
| oLC9753 | *CaENTH Y100R R* | GAACAAATTCTCTTAAAGTTTTAATAATGCGAAGATTGTCTTTGGCCCATAACACACAT |
| oLC9754 | *CaENTH T104D F* | GTTATGGGCCAAAGACAATCTTTACATTATTAAAGATTTAAGAGAATTTGTTCATTTCGATG |
| oLC9755 | *CaENTH T104D R* | CATCGAAATGAACAAATTCTCTTAAATCTTTAATAATGTAAAGATTGTCTTTGGCCCATAAC |
| oLC9758 | *CaENTH R62L F* | GAAGTTATGGATATGTTAGATCGTCTTCTTAATGATAAAGGTAAAAATTGG |
| oLC9759 | *CaENTH R62L R* | CCAATTTTTACCTTTATCATTAAGAAGACGATCTAACATATCCATAACTTC |
| oLC9760 | *CaENTH H72L F* | TAAAGGTAAAAATTGGAGACTTGTGGCCAAATCATTGACGG |
| oLC9761 | *CaENTH H72L R* | CCGTCAATGATTTGGCCACAAGTCTCCAATTTTTACCTTTA |
| oLC9996 | *CaENTH+95F* | ACCCACCACGTTTGATATGG |
| oLC10k657 | FRT guide F | TTCTAGAGCGGCCGCCACCGGTTTTAGAGCTAGAAATAGCAAGTTAAAA |
| oLC10k658 | FRT guide R | CGGTGGCGGCCGCTCTAGAACAAATTAAAAATAGTTTACGCAAGTC |
| oLC9631 | CaEnt2-Arg R | TCGATACATTTGCGGTACAGAAATGTTCTTTTAAATATCTATTAAACTTGG |
| oLC9632 | pLC45-Ent2 F | AATGATGGCCCAAGTTTAATAGATATTTAAAAGAACATTTCTGTACCGC |
| oLC9633 | pLC45-Ent2 R | TGTTGTTGTTGTTATATGCTATCAATATAACATCAATTCTAAAAAGCAATTATTGTTGATTGGAACTTGTACAAAAGCTATTTGCATCG |
| oLC9636 | Ent2-Arg Fusion F | TGTTGTCGTTGTTGCTGAGG |
| oLC9637 | Ent2-Arg Fusion R | AAGCAATTATTGTTGATTGG |
| oLC9687 | pLC45 CdArg4 F | CACACAAGACAAACGATACG |
| oLC9634 | CaENTH-Arg R | TGCGGTACAGAAATGTTCTTTTAATTCTTTTTAGCATTAGCACGCTCTTGTTTTAATCGC |
| oLC9635 | pLC45-ENTH F | ACAAGAGCGTGCTAATGCTAAAAAGAATTAAAAGAACATTTCTGTACCGCAAATGTATCG |
| oLC9008 | NEUT5L-pFA F | GCTCGGAGGAGGCTCCCCAAAGGTTTTATCACCAATGGTGGTACCACTAACCCAAGAACAGAAAAAGCATTGGGTCGACGGATCCCC |
| oLC9009 | NEUT5L-pFA R | CAGAGTATGTGAAGCAATTGCAATTGCAATTATTAGAGATCCAGAAAACTGAATTGTGCTTGAATACCACTCGATGAATTCGAGCTCG |
| oLC6729 | NEUT5L F | ATGCTGAATCACTTGATAGG |
| oLC6730 | NEUT5L R | CACAGCTTATTAACGATTCG |
| oLC10k114 | CaALS3 qRT F | TCCAACAACTGAAAGTGAGGT |
| oLC10k115 | CaALS3 qRT R | TAGAAGTGGAAGCAGCTGTG |
| oLC3796 | CaHWP1 qRT F | CCACTACTACTGAAGCCAAATC |
| oLC751 | CaHWP1 qRT R | AAGTGGATACTGTACCAGTTGG |
